# Post-stroke cognitive impairment: More than a lesion-symptom model

**DOI:** 10.1162/IMAG.a.1050

**Published:** 2025-12-11

**Authors:** Hanne Huygelier, Margaret Jane Moore, Annick Odom, Nora Tuts, Hella Thielen, Mauro Mancuso, Céline R. Gillebert, Nele Demeyere

**Affiliations:** Brain and Cognition, Leuven Brain Institute, KU Leuven, Leuven, Belgium; Experimental Psychology, University of Utrecht, Utrecht, The Netherlands; Queensland Brain Institute, University of Queensland, Brisbane, Australia; Physical and Rehabilitative Medicine Unit, NHS-USL South-East Tuscany, Grosseto, Italy; Tuscany Rehabilitation Clinic, Montevarchi (AR), Montevarchi, Italy; Nuffield Department of Clinical Neurosciences, University of Oxford, Oxford, United Kingdom

**Keywords:** post-stroke cognitive impairment (PSCI), cognitive profiles, latent class analysis (LCA), lesion-symptom mapping, multivariate similarity analysis

## Abstract

Theoretical neuropsychology has traditionally investigated brain–behaviour relationships by measuring the extent to which individual cognitive impairments map onto distinct lesion correlates. However, individual post-stroke cognitive impairments (PSCI) rarely occur in isolation. In the current study, we employ multivariate analysis techniques to explore the extent to which individual, multi-domain patterns of PSCI can be distinguished into distinct cognitive profiles and the extent to which these profiles are associated with lesion anatomy. Latent Class Analysis (LCA) was conducted on domain-specific cognitive screening data from a representative stroke cohort (n = 2172). Voxel-level and network-level lesion mapping was used to identify lesion correlates of identified PSCI profiles. In addition, the association of the PSCI profiles with general brain health (e.g., atrophy severity, white matter integrity), and demographic characteristics was investigated. LCA identified two viable cognitive class solutions: a 5-class model and a 13-class model. The 5-class solution distinguished classical lateralised stroke deficits (e.g., aphasia, neglect) alongside a minimal impairment and non-lateralised global impairment profile. In contrast, the 13-class solution provided finer-grained differentiation, particularly for non-lateralised cognitive profiles which were more strongly associated with premorbid health and education level. Importantly, lesion anatomy alone could not fully account for class distinctions. While lesion location was predictive, particularly, in hyper-acute stages, profiles for patients tested 2 weeks post-stroke revealed less influence of lesion location and more of lesion volume. These findings provide a novel, multivariate conception of PSCI, which establishes the theoretical groundwork necessary to support future translational research aimed at improving clinical care and predicting cognitive trajectories.

## 1 Introduction

Cognitive impairment is a common consequence of stroke which is associated with poor recovery and outcomes in daily life (Stolwyk et al., 2021). Post-stroke cognitive impairment (PSCI) does not impact all cognitive skills equally, but instead manifests as a diverse range of domain-specific cognitive impairments which may selectively impact a wide range of cognitive functions including language, memory, and attention (Demeyere et al., 2016). While theoretical neuropsychology has traditionally investigated brain–behaviour relationships by measuring the extent to which individual cognitive impairments map onto distinct lesion correlates, the clinical reality is that individual PSCI deficits rarely occur in isolation. Indeed, PSCI typically involves co-occurring impairments across multiple cognitive domains (Bickerton et al., 2015; Demeyere et al., 2015; Nys et al., 2007; Tatemichi et al., 1994). Past research has demonstrated that patterns of cognitive associations (and dissociations) vary widely across individuals, but the extent to which this behavioural variability may be captured by a reduced set of underlying factors is not yet clear (Contador et al., 2023; Hobden et al., 2023; Stebbins et al., 2008). This question is both theoretically and clinically relevant, as exploring the factors underlying individual variability in PSCI can provide novel insight into complex brain–behaviour relationships while also establishing a novel, multi-domain cognitive framework which can be applied to better understand the cognitive needs of stroke patients.

Past research employing Principal Component Analysis (PCA) has suggested that individual variability in multi-domain PSCI can be at least partially explained by a reduced set of underlying behavioural factors (i.e., “dimensions”). Corbetta et al. (2015) evaluated language, motor, memory, and attention in first-time stroke survivors (n = 67) to identify factors explaining behavioural correlations across subjects. This study identified 3 factors (1 lateralised to each hemisphere and a non-lateralised factor) accounting for 69% of behavioural variance (Corbetta et al., 2015). Similarly, Bisogno et al. (2021) found that approximately 50% of variance on the Oxford Cognitive Screen (OCS), a domain-specific cognitive screen for stroke patients, could be accounted for by 3 factors. The first factor captured language, calculation, praxis, right-lateralised spatial neglect, and memory. The second factor loaded on left motor and visuospatial deficits, and the third factor loaded on right motor impairment (Bisogno et al., 2021). However, a more recent larger-scale (n = 1973) PCA found that OCS performance was best captured by a six-factor solution (language/arithmetic, memory, visuomotor ability, orientation, spatial exploration, and executive functions) (Iosa et al., 2022). Overall, past research has suggested that the variability in PSCI may be captured by a reduced number of dimensions, but the number and underlying nature of these dimensions have not been reliably established.

While PCA has been useful in describing patterns of PSCI associations, this approach may underestimate the complexity of PSCI (Sperber et al., 2022). Sperber et al. (2022) demonstrated that the systematic spatial variability of stroke lesion anatomy alone is sufficient to result in an apparent low-dimensional structure underlying PSCI, even when all simulated impairments were independent. Consequently, Sperber et al. (2022) called for future studies to develop and compare latent structures of varying complexity, suggesting that additional factors which do not increase explained variance but produce an intuitively interpretable solution should be retained in solutions. Latent Class Analysis (LCA) is an analytical approach that can address this research gap. While PCA aims to explain covariance using a restricted set of continuous dimensions that represent shared cognitive mechanisms (e.g., a visual dimension explaining praxis and picture naming performance), LCA explains the covariances through distinct subpopulations which differ qualitatively (Lubke & Muthén, 2005). An LCA model can identify stroke subpopulations characterised by specific comorbidities which arise from co-occurring damage to independent cognitive functions. LCA enables the identification of such “subpopulations”, for which PCA is not ideal (Sperber et al., 2022). However, LCA also has some limitations. For example, if tests are correlated due to overlap in what they measure, LCA models may overestimate the number of subpopulations (Lubke & Muthén, 2005). LCA (and PCA) both explain covariance based on a single source, and it is likely that a combination of shared cause and independent co-occurrence explains PSCI profiles. However, in cases where the extensive behavioural data (e.g., data from several tests per function of interest) needed to support joint modelling of both sources of covariance (i.e., factor mixture models; Lubke & Muthén, 2005) are not available, LCA still provides a powerful method for identifying distinct profiles of PSCI impairment (Porcu & Giambona, 2017).

It is also important to consider that profiles of lesion-induced disconnection, in addition to lesion location, may help explain patterns of PSCI associations. Past work has demonstrated that individual PSCI domain impairments can be mapped onto distinct patterns of network-level disconnection (Bowren et al., 2022; Moore et al., 2024), but the extent to which these disconnection patterns may help account for common PSCI profiles remains unclear.

However, it is important to recognise that lesion anatomy alone is unlikely to fully account for the variability in PSCI. This is because the relationship between impairments and lesion anatomy is often confounded by pre-existing neurovascular changes (Rost et al., 2022). Premorbid factors including cerebral atrophy (Casolla et al., 2019; Stebbins et al., 2008), white matter integrity (Dacosta-Aguayo et al., 2014; de Kort et al., 2023), and education level (Contador et al., 2023; Umarova et al., 2019) are each associated with an increased risk of PSCI. There is also high comorbidity between stroke and major neurocognitive disorders and mild cognitive decline (Bunn et al., 2014). These brain health markers are typically associated with impairment in memory, executive functions, processing speed, and language (de Kort et al., 2023; Demeyere et al., 2021; Yanhong et al., 2013), which can occur alongside stroke-related cognitive impairments. The latter complicates the clinical picture of PSCI.

Although many studies have documented the importance of these factors for PSCI, there is a lack of studies investigating across-domain PSCI profiles and how such profiles link to lesion anatomy and premorbid brain health. That is, prior studies using data-driven strategies to investigate the structure of PSCI have either focused on separate cognitive domains (Bonkhoff et al., 2021; Landrigan et al., 2021; Weaver et al., 2023), a global cognition outcome (Buvarp et al., 2021; Keins et al., 2021; Ma et al., 2024; Weaver et al., 2021), or have focused on a select group of stroke patients (such as first-ever stroke patients with good premorbid brain health) (Buvarp et al., 2021; Corbetta et al., 2015; Keins et al., 2021; Ma et al., 2024). Given the high comorbidities in the clinical reality of stroke, it is important to disentangle distinct cognitive profiles and investigate to what extent such profiles align with specific lesion topographies versus pre-existing neurovascular changes, especially in a clinical stroke sample.

The present study seeks to address this gap by using latent class analysis to investigate cognitive profiles in a large stroke cohort assessed with the Oxford Cognitive Screen (OCS). This approach allows to explore the structure of PSCI in a clinically representative stroke sample beyond the limitations of global cognitive screens (Demeyere et al., 2016). Specifically, our objectives were to (1) distinguish cognitive profiles after stroke, (2) assess and compare the interpretability of these profiles, and (3) evaluate the extent to which stroke lesion anatomy, premorbid brain health, and demographic variables influence the structure of PSCI.

## 2 Materials and Methods

Data of 2172 stroke patients were aggregated from Belgian, Italian, and UK databases (Demeyere et al., 2015, 2016, 2019; Huygelier et al., 2022; Mancuso et al., 2018). These studies included all patients who were able to concentrate for 15–20 minutes (Supplementary Materials S1).

### 2.1 Ethics statement

All patients provided informed consent and all procedures followed the Helsinki declaration.

### 2.2 Cognitive screening

Domain-specific impairments were assessed with three language versions of the Oxford Cognitive Screen (i.e., OCS, OCS-IT, OCS-NL) (Demeyere et al., 2015, 2016; Huygelier et al., 2020, 2022; Mancuso et al., 2016, 2018). These OCS translations have each been validated in stroke patients (Demeyere et al., 2015; Huygelier et al., 2022; Mancuso et al., 2018). The OCS is designed to be inclusive for patients with common stroke-related impairments including aphasia, spatial neglect, primary visual, and motor impairments (Demeyere et al., 2015, 2016). The OCS consists of 10 subtasks indexing: language (picture naming, semantics, sentence reading), memory (orientation, verbal, and episodic memory), numerical cognition (writing numbers, calculation), praxis (imitating meaningless gestures), and executive function (trail making)/attention (cancellation test). Detailed descriptions of OCS subtests are reported elsewhere (Demeyere et al., 2015, 2016). All impairment classifications were made based on clinical thresholds which were age corrected for the OCS-NL (Huygelier et al., 2020) and OCS-IT (Mancuso et al., 2016).

### 2.3 Neuroimaging data

All patients with lesion masks and behavioural data collected within 45 days of stroke onset were included in neuroimaging analyses (n = 515). Lesion maps were derived from acute (<31 days post-stroke) clinical neuroimaging (442 CT, 4 T1, 62 T2, 6 FLAIR) which were collected in the UK OCS screening programmes (Demeyere et al., 2015, 2016). Previous research has demonstrated that neuroimaging collected within 31 days of stroke is able to support accurate lesion mapping analyses, even though the full extent of lesion damage may not yet be fully visible at very early (e.g., <1 day) imaging time points (Lansberg et al., 2000; Mohr et al., 1995; Moore et al., 2024). Previous studies have found that CT and MR yield comparable lesion mapping results and that both imaging modalities can be used to accurately identify established PSCI correlates (de Haan & Karnath, 2018; Moore, Demeyere et al., 2024; Moore, Jenkinson et al., 2023). Notably, a recent, large-scale simulation study (using a subset of the data reported here) compared the accuracy of lesion mapping analyses using CT- and MR-derived lesion masks. This study found that overall accuracy was low for both CT- and MR-derived analyses, but analyses using CT data were significantly more accurate in terms of target hits (e.g., detecting the critical voxel/area) and in terms of displacement from the target area/voxel relative to MR-based analyses (Moore, Jenkinson et al., 2023). In addition to this previous lesion mapping, guidelines and manuals explicitly encourage combining different imaging modalities (CT and MR) to maximise the degree of overlap (i.e., statistical power) in lesion mapping (de Haan & Karnath, 2018). Patients with clear evidence of multiple, temporally distinct lesions were excluded.

Lesions were manually delineated by trained experts on axial slices using MRIcron. Native-space lesion masks were smoothed at 5 mm full-width at half-maximum in the z-direction, binarised (0.5 threshold), reoriented, warped into 1 × 1 × 1 mm stereotaxic space using Statistical Parametric Mapping (Ashburner et al., 2016) and Clinical Toolbox (Rorden et al., 2012) functions. This normalisation approach uses combined linear and affine transformations coupled with nonlinear warping to Clinical Toolbox templates (Ashburner et al., 2016; Rorden et al., 2012) (scripts available at https://osf.io/mv2qf/files/osfstorage). All resulting normalised scan and lesion files were visually inspected for quality. This normalisation procedure employs age-specific CT or MR templates (Rorden et al., 2012). Exploratory analyses were conducted to ensure behavioural results were consistent between the total sample and subset of patients with lesion data (Supplementary Materials, Supplementary Figs. S3.1 and S3.2).

To estimate the degree of disconnection within functional networks caused by structural lesion damage, lesion masks were used to estimate the degree of disconnection between different cortical (and subcortical) areas caused by lesion damage. This approach is standard in cases where in vivo tractography data are not available, and are described in detail elsewhere (Griffis et al., 2021). Parcel-wise dysconnectivity statistics were generated to summarise the degree of disconnection across all cortical areas defined by the Schaefer–Yeo Atlas (100 parcels) (Yeh et al., 2018) and subcortical/cerebellar areas in the AAL (Tzourio-Mazoyer et al., 2002) and Harvard–Oxford atlases (35 parcels). The atlases define seven networks: Control, Default, Dorsal Attention, Limbic, Somatic Motor, Ventral Attention, and Visual networks as well as networks connecting subcortical/cerebellar structures (Schaefer et al., 2018). In cases where lesions intersect with a streamline (i.e., a connection between two parcels), the relevant streamline is considered to be disconnected (Griffis et al., 2021). Network nodes correspond to each defined cortical areas, and network edges summarise the connections (streamlines) between each pair of nodes (Griffis et al., 2021). Network-level disconnectivity was calculated as the proportion of streamlines which terminate (end or begin) in each pair of parcels that were disconnected (Griffis et al., 2021). Tract-level structural disconnection was quantified by calculating the percent of streamlines within each of the HCP-842’s 70 white matter tracts which were disconnected by lesions.

Atrophy was assessed using the Global Cortical Atrophy (GCA) scale (Pasquier et al., 1996). The atrophy in 13 brain regions was assigned a score of 0 (none), 1 (mild), 2 (moderate), or 3 (severe). Where regions were obscured by stroke lesions, regions were assigned the score of the homologous region within the undamaged hemisphere. The severity of white matter lesions was determined using the Fazekas scale (Fazekas et al., 2002). Deep white matter lesions were rated as 0 (absent), 1 (punctuate foci), 2 (beginning confluence of foci), or 3 (large confluent areas). Periventricular white matter lesions were rated as 0 (absent), 1 (symmetrical caps), 2 (smooth halo), or 3 (irregular hypoattenuations extending into deep white matter). Total GCA and Fazekas scores (n = 475) were calculated by adding all region scores (GCA range = 0–39, Fazekas range = 0–6). Both scales have been validated for use in CT (Hobden et al., 2024) and MR stroke imaging (Fazekas et al., 2002; Pasquier et al., 1996). Given that Fazekas and GCA scores are derived from imaging, these measures were only available for patients with neuroimaging data.

### 2.4 Statistical analyses

Data analysis was performed using R 4.0.2, python 3.9, and MATLAB R2022b. The specific packages employed in each analysis are reported in Supplementary Materials S2 and S3.

#### 2.4.1 Identifying distinct PSCI profiles

Latent class analysis (LCA) was used to identify common profiles of PSCI in a data-driven fashion. LCA assumes that the associations of the observed data arise from unobserved subgroups (i.e., classes) in the population. LCA is more powerful than k-means or hierarchical clustering (Cleland et al., 2000; Porcu & Giambona, 2017), as it enables comparisons of models of different numbers of classes (Nylund-Gibson & Choi, 2018) and because it allows for class membership uncertainty (estimating the probability that an individual belongs to each identified class).

To establish the classes, only the binary impairment classifications on OCS subtests were included as observed variables (Bakk & Kuha, 2021). The LCA models used 10 repetitions (maximum of 50,000 iterations each) with different random starting values. The models were estimated including all partially available cases (Enders & Bandalos, 2001; Linzer & Lewis, 2011). To determine the ideal number of classes, models with n and n + 1 classes were compared with the Bayesian Information Criterion (BIC), Akaike Information Criterion (AIC), and the adjusted Lo–Mendell–Rubin likelihood ratio test (Lo et al., 2001; Nylund-Gibson & Choi, 2018; Petersen et al., 2019; Tein et al., 2013). A difference in the BIC and AIC larger than 2 is considered substantial evidence in favour of the more complex model (Burnham & Anderson, 2004). To investigate class distinguishability, the relative entropy (ranging from 0 = no separation to 1 = perfect separation) was calculated (Celeux & Soromenho, 1996).

#### 2.4.2 Interpretation of PSCI profiles

To interpret LCA classes, the cognitive profiles of patients in each behavioural class were summarised. Class members were always compared with non-members to identify unique characteristics of each class. Specifically, each class’s probability of impairment and 95% confidence interval for each subtest were compared with that of patients belonging to other classes. Each class was assigned a summary label providing a qualitative description of the most prevalent impairment types within each class. This label is used to aid referencing and interpretation but is not intended as a formal definition.

Neuroanatomical characteristics were compared between class members and non-members. First, we estimated differences in proportions of left- and right-hemispheric patients. Second, the ratio of lesion volume between class members and non-members was estimated. Third, differences in GCA and Fazekas scores were estimated. In addition, differences in proportions of class members versus non-members in different age groups (≤60: 22% of patients, 61–80: 50% of patients, and >80: 28% of patients), education levels (<7: 17% of patients, 7–12: 54% of patients, and >12 years of schooling: 29% of patients), stroke chronicity groups (<3 weeks: 73% of patients, ≥3 weeks post-stroke: 27% of patients), and sex were estimated. Model fits were satisfactory and all modelling details are reported in Supplementary Materials S2.

Mass-univariate voxel-based lesion analyses (VLSM) were conducted to evaluate whether classes were characterised by distinct lesion profiles. Each of these analyses included lesion volume as a covariate of no interest and employed permutation-based thresholds (2000 permutations) to correct for multiple comparisons (alpha = .05) (de Haan & Karnath, 2018). These analyses were performed with NiiStat (neurolabusc, 2016) and only considered voxels which were impacted in at least 10 patients (Supplemental Materials S2). Resultant significant voxels clusters (>10 contiguous voxels) were compared with anatomical atlases.

We also contrasted network disconnections of class members versus non-members, using a test that assumes that edges that differ between groups are clustered into components (i.e., a subnetwork of connected edges) (Gracia-Tabuenca & Alcauter, 2020). This approach increases statistical power relative to mass-univariate statistical comparisons (Kim et al., 2014; Zalesky et al., 2010) by controlling the family-wise error at the subcomponent rather than edge level. Following the NBS procedure, we first identified a set of edges which had the strongest associations with class membership (supra-threshold edges) (uncorrected p < .01). To evaluate the quality of identifying our supra-threshold edges, we checked the differences in edge weights for the supra- and sub-threshold edges (Supplementary Materials S3). Then, these edges are entered into a permutation-based test (1000 permutations) that identifies statistically significant components (i.e., subnetwork of connected edges), estimating an FWE-corrected p-value for the sum of edge weights of the component. Then, we quantified *network disconnection*. An edge was considered part of a functional network if at least one node belonged to the network (e.g., both inter- and intra-network connections) (Supplementary Materials S3). The VLSM and network analyses were only conducted for classes which contained at least 15 patients with available lesion data.

#### 2.4.3 Does lesion topography drive PSCI profiles?

Finally, we evaluated the extent to which lesion anatomy drove the LCA solution using multivariate similarity analysis. Multivariate similarity analysis is a popular neuroimaging analysis technique (Kriegeskorte & Kievit, 2013), which we applied here to determine to what extent patients who were behaviourally similar were also similar in terms of lesion anatomy. This analysis is an informative precursor to analyses aiming to predict specific behavioural profiles based on lesion data alone (or vice versa). If multivariate similarity analysis reveals that there is a high degree of similarity between lesion topographies within behavioural cluster members (relative to non-members), this indicates that it may be feasible to accurately predict profile membership based on lesion anatomy. However, if similarity analysis finds weak or negligible relationships between anatomy and profile membership, this indicates that lesion anatomy alone cannot provide an informative prediction of profile membership.

**Fig. 1.**
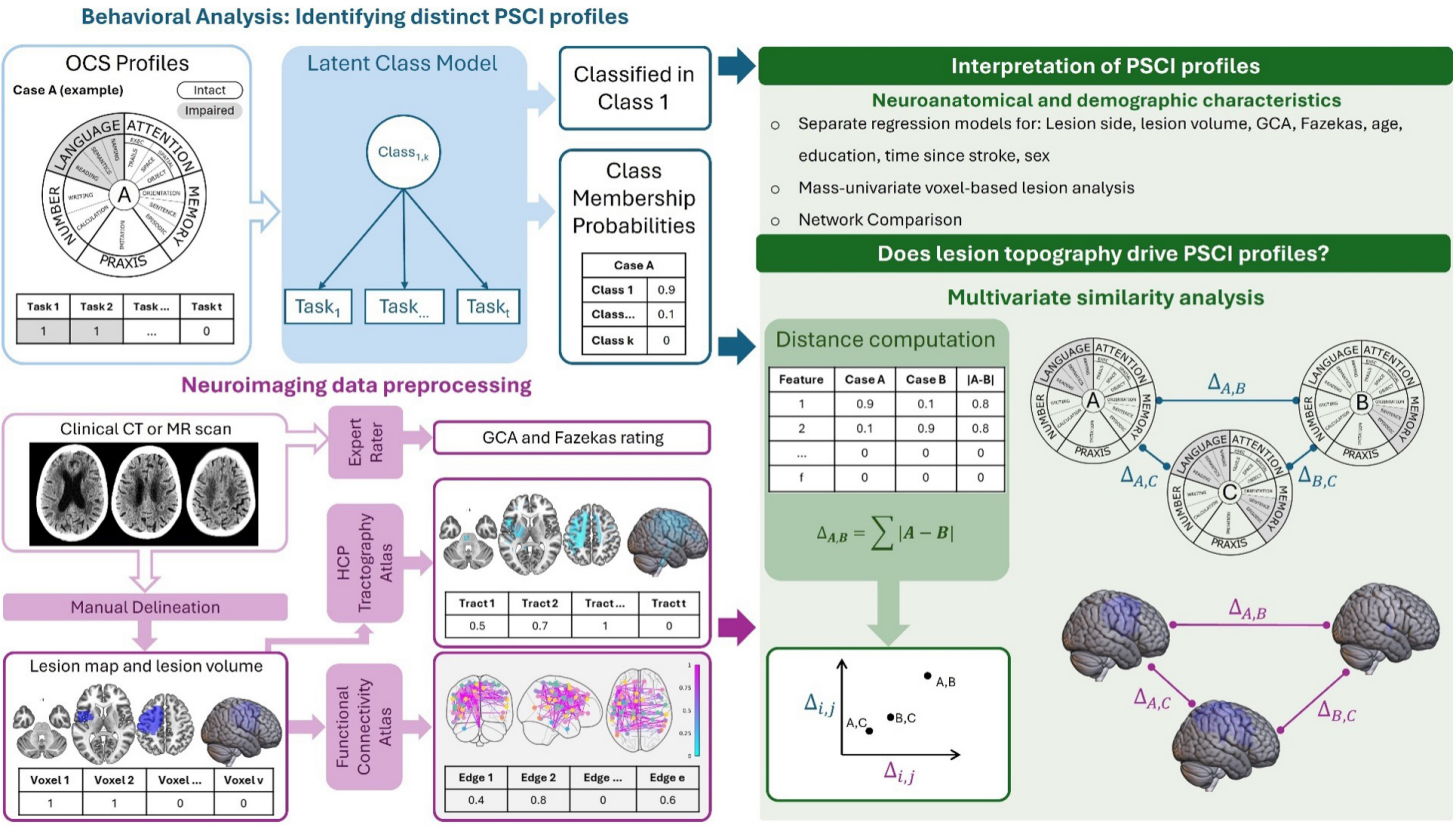
Overview of the analysis. Top left (blue) illustrates an OCS profile of a single case, the LCA model, and output we obtain for case A from the LCA model. Bottom left (purple) illustrates preprocessing of clinical CT or MR scans. The right side (green) illustrates the association of cognitive profiles, neuroanatomy, and demographic variables. For the multivariate similarity analysis, a distance is computed for each pair of cases (example of three cases) and the association of cognitive and neuroanatomical distances was then calculated.

In our analysis, multivariate similarity analysis was used to determine the degree to which similarity in terms of profile membership (cognitive similarity) was associated with similarity in terms of lesion topography/disconnection (neuroanatomical similarity). To quantify cognitive similarity, we computed the distance between the class probabilities for each pair of participants. We also computed neuro-anatomical (dis)similarity at the voxel, tract, and network levels. Then we assessed the association between the cognitive and the neuroanatomical distances (Fig. 1; Supplementary Materials S3).

The robustness of the similarity analysis was evaluated across key patient subgroups. For example, premorbid brain health decline may reduce the association between lesion location and the cognitive profile (Rost et al., 2022). In addition, haemorrhagic stroke patients can suffer from diffuse damage meaning that the relationship between cognition and lesion location could be weaker in this subgroup (Sheppard et al., 2022). Given that previous research has suggested that lesion location–behaviour relationships are strongest early after stroke onset, we also evaluated how this association depended on time after stroke (de Haan & Karnath, 2018; Sheppard et al., 2022). For all associations, we performed a leave-one-case-out sensitivity analysis which indicated robust results across subsamples.

## 3 Results

### 3.1 Participants

**Fig. 2.**
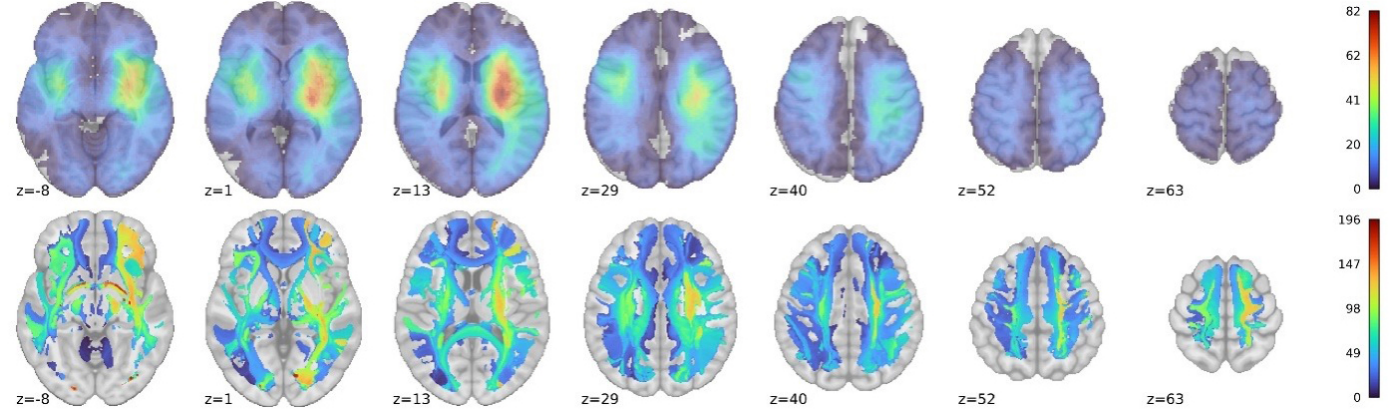
Lesion overlay and tract disconnections (n = 515). Number of patients with ≥50% disconnection in a tract. MNI slices -8–63 are presented in neurological convention (right hemisphere presented on the right side).

Patients were assessed on average 19 days post-stroke (*Mdn* = 7, *SD* = 35.7). A total of 43% of patients had a left-hemispheric stroke, 50% had a right-hemispheric stroke, and 7% had a bilateral stroke. Most patients had an ischaemic stroke (81% ischaemic, 19% haemorrhage) and 45% were female. The average age of the patients was 71 years (*SD* = 13.6). The average years of formal education was 11 years (*SD* = 4). The lesion distribution and tract disconnections of patients with a lesion map are visualised in Figure 2. Participant demographics, including testing times and lesion descriptions, are reported in Table 1.

**Table 1.** Patient characteristics by country.

		UK (n = 1206)	IT (n = 684)	BE (n = 282)
Age	M	72.4	71.1	64.8
	Mdn	75	73	67
	SD	13.6	12.8	14
	Min-Max	18–98	24–96	21–91
Years of education	M	11.7	8.6	12.4
	Mdn	11	8	12
	SD	2.9	4.5	3.6
	Min-Max	5–30	1–25	5–25
Sex	% Female	45%	45%	39%
Days since stroke	M	8.7	37.6	22.7
	Mdn	4	20	15
	SD	14.4	54.7	24.4
	Min-Max	0–208	0–567	0–182
Stroke type	% Ischaemic	84%	77%	81%
Lesion side	% Left	46%	40%	38%
	% Right	48%	56%	44%
	% Bilateral	5%	4%	18%

### 3.2 PSCI can be distinguished into 5 or 13 profiles

**Fig. 3.**
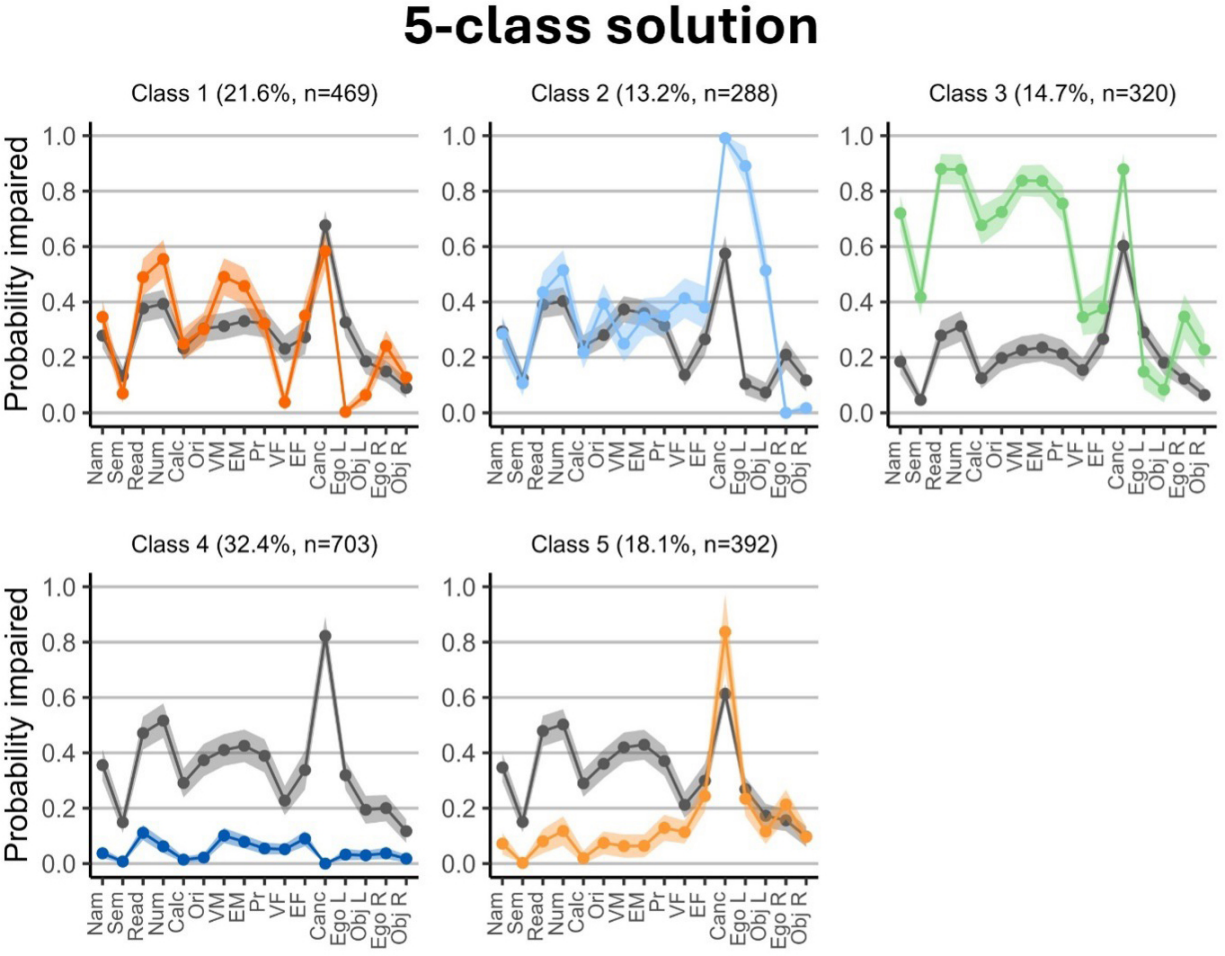
Cognitive profiles of the 5-class model. The probability of impairment per OCS subtest and its 95% confidence interval are depicted of class members (colour) versus others (grey). Nam = OCS picture naming task, Sem = OCS semantics tests, Read = OCS sentence reading task, Num = OCS number writing task, CAL = OCS calculation task, ORT = OCS orientation task, VM = OCS verbal memory task, EM = OCS episodic memory task, PR = OCS praxis test, Canc = OCS cancellation total score, Ego L = OCS left egocentric neglect, Obj L = OCS left object-level neglect, Ego R = OCS right egocentric neglect, Obj R = OCS right object-level neglect.

**Fig. 4.**
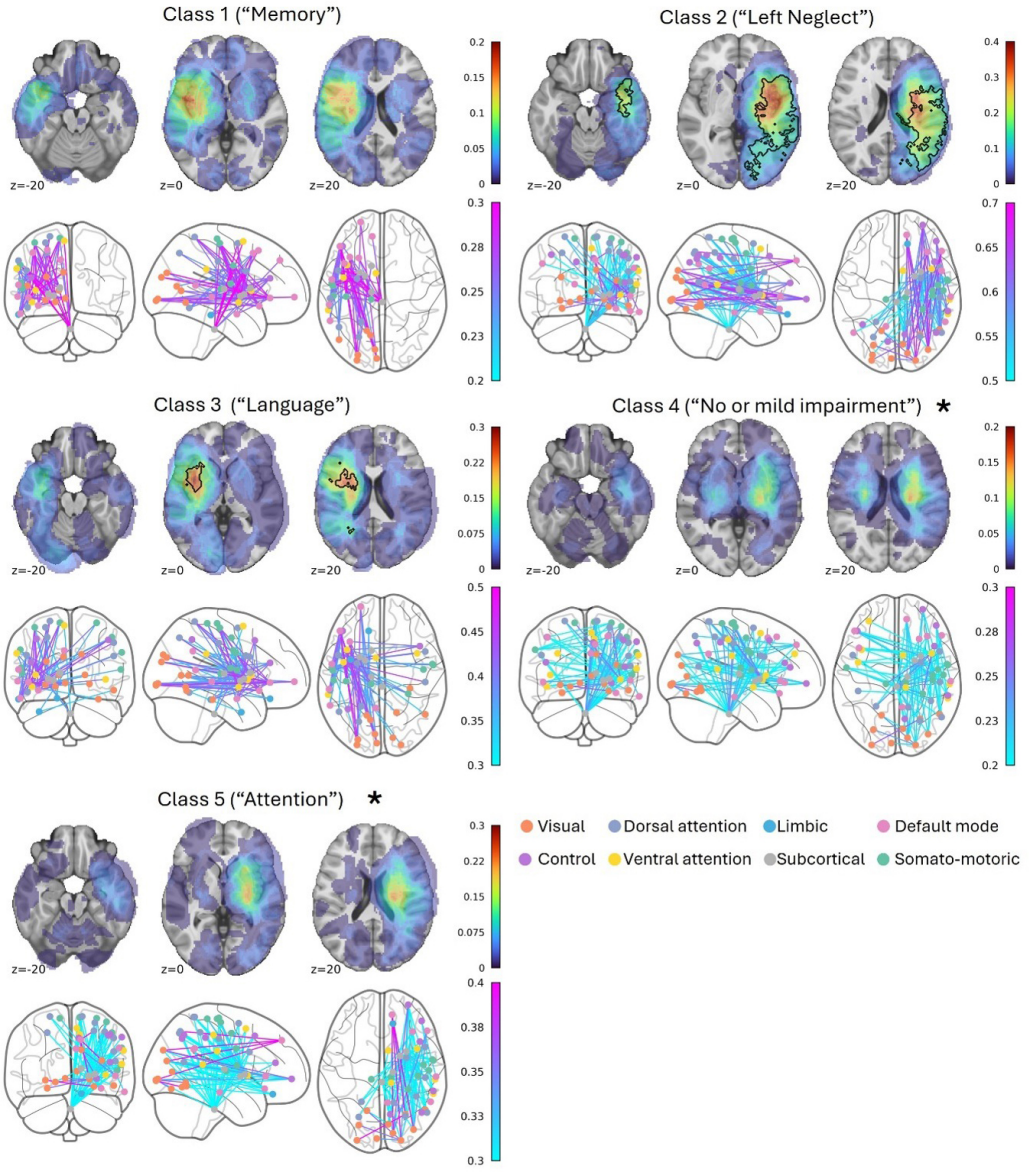
Lesion correlates of the 5-class model. Top rows depict the lesion overlay (colours represent the proportion patients in a class with a lesion at a specific voxel). A black contour indicates a region of significant voxels. The Bottom row: 1% strongest network-level disconnections which were part of a statistically significant component of edges. *Non-significant strongest disconnections depicted. The colour of the edges represents the mean proportion disconnection of class members. Neuroimaging is presented in neurological convention.

**Fig. 5.**
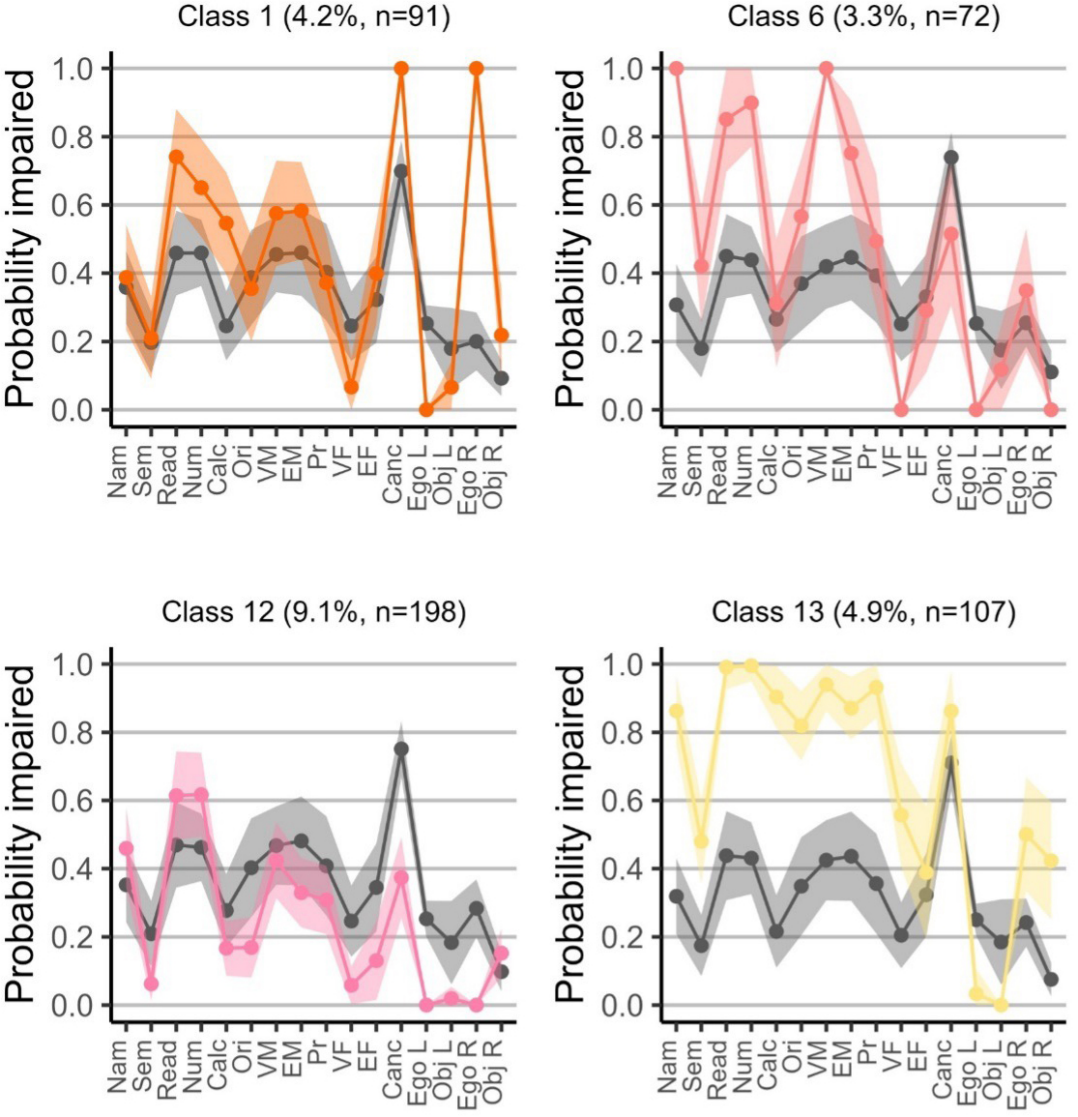
Cognitive profiles of the left-lateralised profiles of the 13-class model. The probability of impairment per OCS subtest and its 95% confidence interval are depicted of class members (colour) versus others (grey). Nam = OCS picture naming task, Sem = OCS semantics tests, Read = OCS sentence reading task, Num = OCS number writing task, CAL = OCS calculation task, ORT = OCS orientation task, VM = OCS verbal memory task, EM = OCS episodic memory task, PR = OCS praxis test, Canc = OCS cancellation total score, Ego L = OCS left egocentric neglect, Obj L = OCS left object-level neglect, Ego R = OCS right egocentric neglect, Obj R = OCS right object-level neglect.

**Fig. 6.**
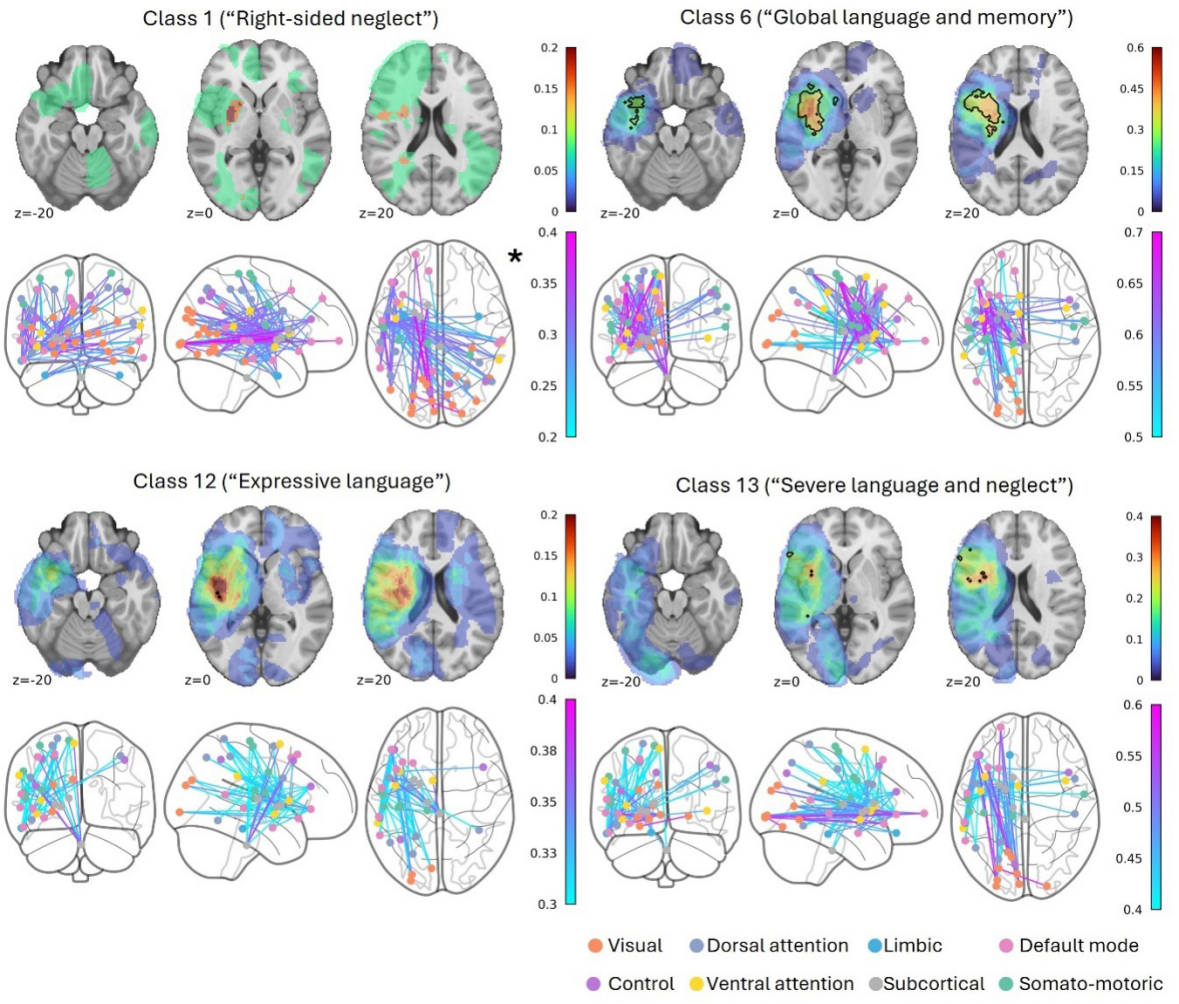
Lesion correlates of the left-lateralised profiles of the 13-class model. Top rows depict the lesion overlay (colours represent the proportion patients in a class with a lesion at a specific voxel). A black contour indicates a region of significant voxels. The Bottom row: 1% strongest network-level disconnections which were part of a statistically significant component of edges. *Non-significant strongest disconnections depicted. The colour of the edges represents the mean proportion disconnection of class members. Neuroimaging is presented in neurological convention.

**Fig. 7.**
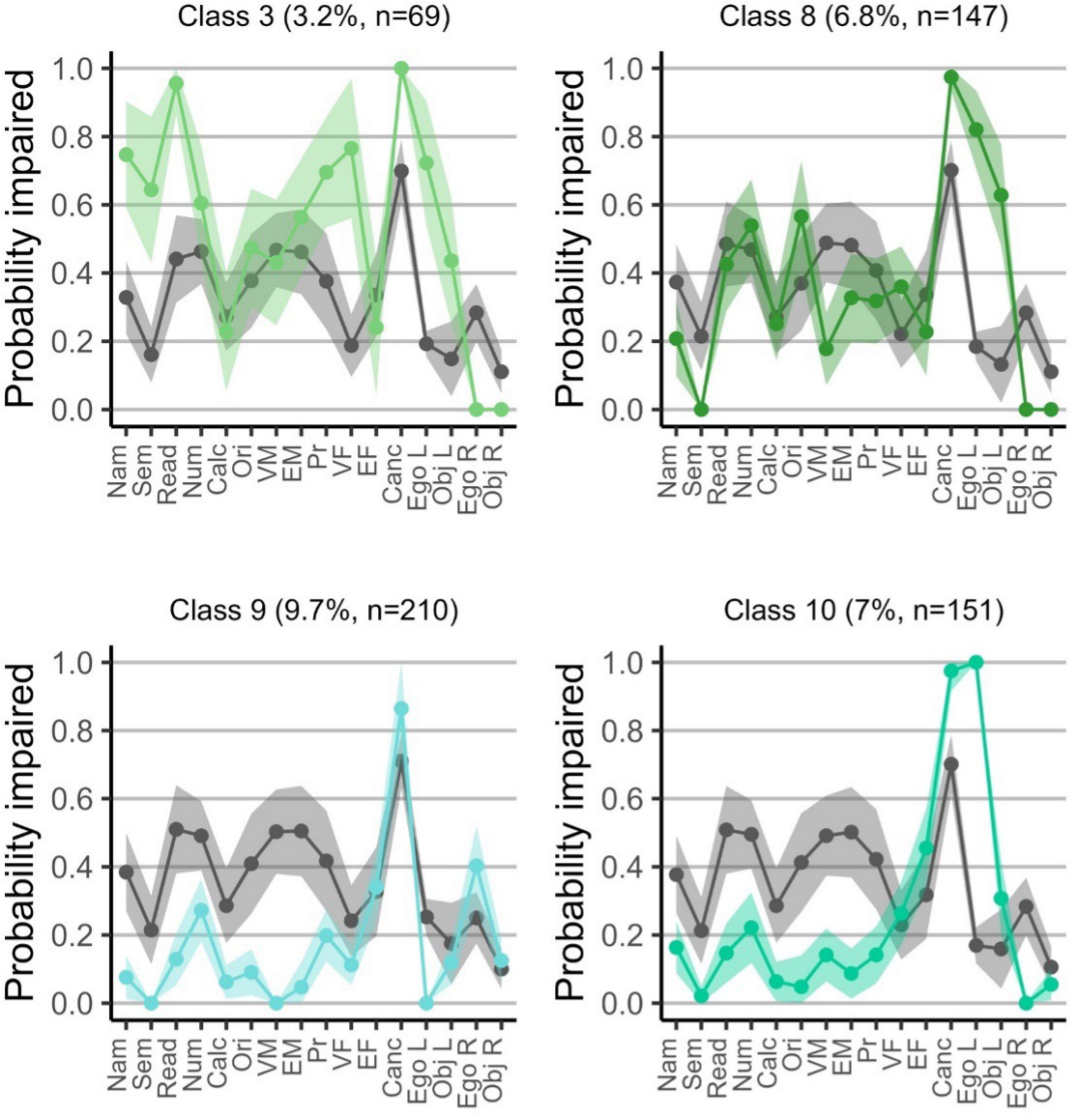
Cognitive profiles of the right-lateralised profiles of the 13-class model. The probability of impairment per OCS subtest and its 95% confidence interval are depicted of class members (colour) versus others (grey). Nam = OCS picture naming task, Sem = OCS semantics tests, Read = OCS sentence reading task, Num = OCS number writing task, CAL = OCS calculation task, ORT = OCS orientation task, VM = OCS verbal memory task, EM = OCS episodic memory task, PR = OCS praxis test, Canc = OCS cancellation total score, Ego L = OCS left egocentric neglect, Obj L = OCS left object-level neglect, Ego R = OCS right egocentric neglect, Obj R = OCS right object-level neglect.

**Fig. 8.**
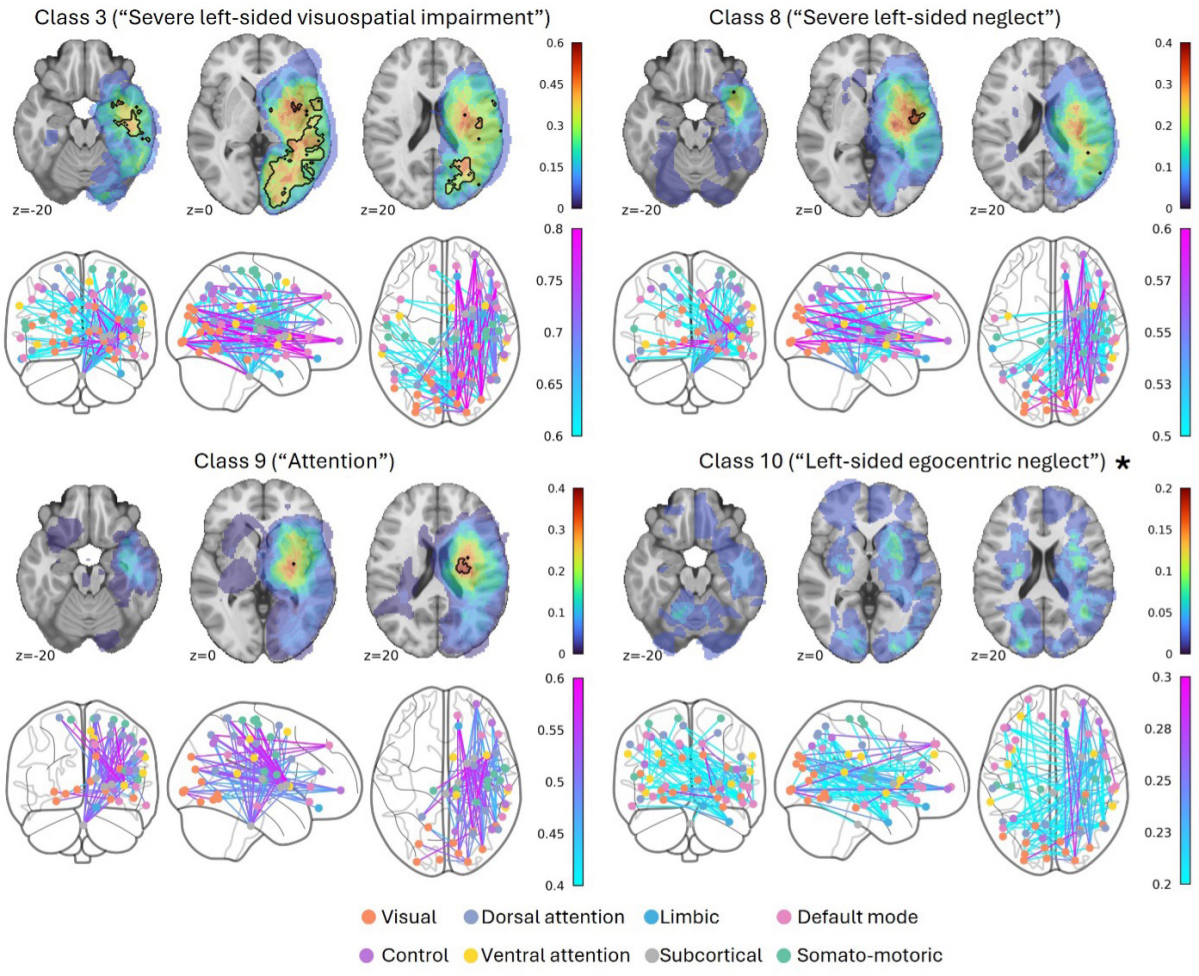
Lesion correlates of the right-lateralised profiles of the 13-class model. Top rows depict the lesion overlay (colours represent the proportion patients in a class with a lesion at a specific voxel). A black contour indicates a region of significant voxels. The bottom row: 1% strongest network-level disconnections which were part of a statistically significant component of edges. *Non-significant strongest disconnections depicted. The colour of the edges represents the mean proportion disconnection of class members. Neuroimaging is presented in neurological convention.

**Fig. 9.**
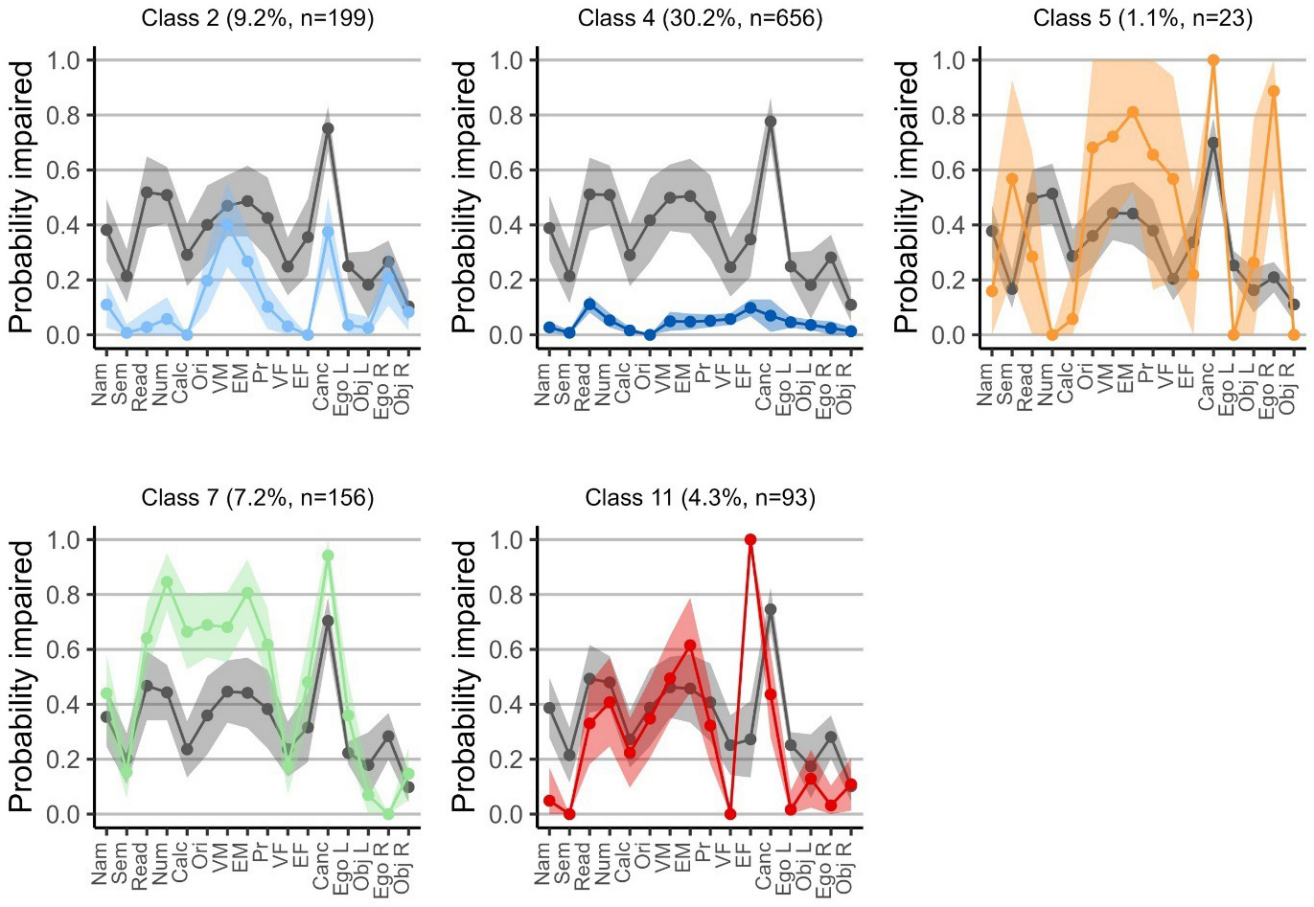
Cognitive profiles of the non-lateralised profiles of the 13-class model. The probability of impairment per OCS subtest and its 95% confidence interval are depicted of class members (colour) versus others (grey). Nam = OCS picture naming task, Sem = OCS semantics tests, Read = OCS sentence reading task, Num = OCS number writing task, CAL = OCS calculation task, ORT = OCS orientation task, VM = OCS verbal memory task, EM = OCS episodic memory task, PR = OCS praxis test, Canc = OCS cancellation total score, Ego L = OCS left egocentric neglect, Obj L = OCS left object-level neglect, Ego R = OCS right egocentric neglect, Obj R = OCS right object-level neglect.

**Fig. 10.**
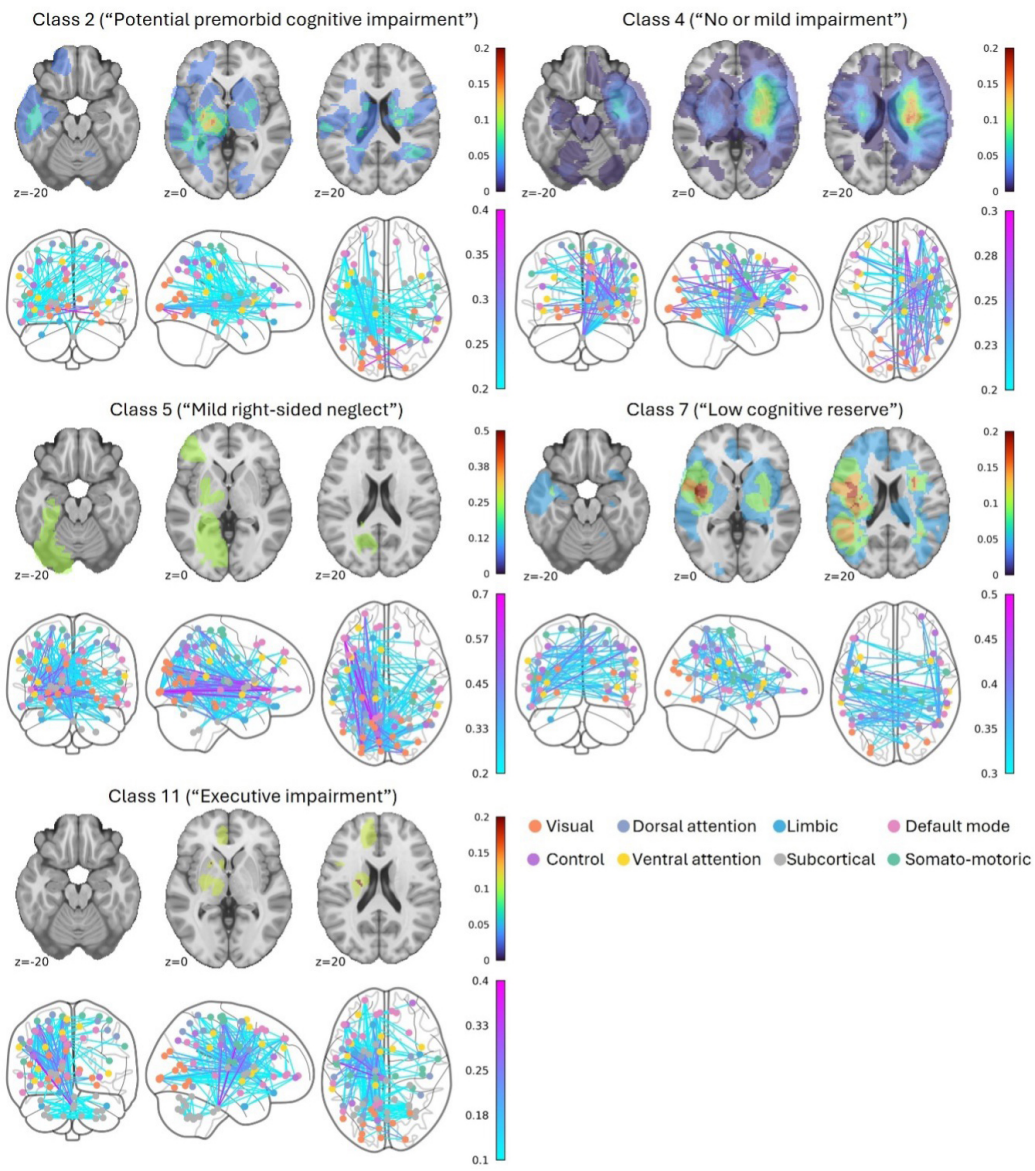
Lesion correlates of the non-lateralised profiles of the 13-class model. Top rows depict the lesion overlay (colours represent the proportion patients in a class with a lesion at a specific voxel). A black contour indicates a region of significant voxels. The Bottom row: non-significant strongest disconnections depicted. The colour of the edges represents the mean proportion disconnection of class members. Neuroimaging is presented in neurological convention.

Two candidate models of distinct cognitive profiles were retrieved: a 5-class model (best fitting according to the BIC index) and a 13-class model (best fitting according to the AIC and Likelihood Ratio Test) (Supplementary Materials S2). The relative entropy was .64, suggesting that there is a moderate level of class separability. The probability that an individual was a member of their respective class was on average 84% for the 5-class model (SD = 16) and 74% for the 13-class model (SD = 19). The probability that an individual was a member of another class was on average 4% for the 5-class model (SD = 7) and 2% for the 13-class model (SD = 4). The relationship between the two solutions is complex, with some classes mapping better onto each other than others across the solutions (Supplementary Fig. S2.6). To interpret the models, we inspected the probability of impairment per OCS subtest for class members versus non-members and their neuroanatomical and demographic characteristics (Figs. 3–10; Tables 2 and 3).

**Table 2.** Characteristics of the 5-class solution.

		Class 1	Class 2	Class 3	Class 4	Class 5
Lesion Side (Proportion)	L	**0.18,** **[0.10, 0.25]**	**-0.36,** **[-0.42, -0.29]**	**0.24,** **[0.15, 0.33]**	**0.09,** **[0.03, 0.15]**	**-0.15,** **[-0.23, -0.07]**
	R	**-0.16,** **[-0.24, -0.08]**	**0.35,** **[0.27, 0.41]**	**-0.22,** **[-0.30, -0.13]**	**-0.10,** **[-0.16, -0.03]**	**0.14,** **[0.05, 0.22]**
	B	-0.02, [-0.05, 0.02]	0.02, [-0.03, 0.08]	-0.03, [-0.06, 0.02]	0.01, [-0.02, 0.05]	0.01, [-0.03, 0.06]
Lesion Volume		0.72, [0.35, 1.43]	**3.57,** **[1.49, 7.69]**	**2.11,** **[1.05, 4.87]**	**0.4,** **[0.22, 0.74]**	1, [0.44, 2.08]
Stroke Time (Acute)		0, [-0.06, 0.07]	0, [-0.09, 0.07]	-0.05, [-0.13, 0.03]	0.03, [-0.03, 0.08]	0.02, [-0.05, 0.09]
GCA		**0.16,** **[0.09, 0.22]**	**0.28,** **[0.19, 0.36]**	0.02, [-0.08, 0.12]	**-0.25,** **[-0.30, -0.19]**	-0.02, [-0.09, 0.04]
Fazekas		0.03, [-0.17, 0.2]	**0.38,** **[0.17, 0.58]**	-0.01, [-0.27, 0.26]	**-0.25,** **[-0.42, -0.11]**	0.05, [-0.14, 0.25]
Age	18–60	-0.06, [-0.11, 0]	-0.01, [-0.08, 0.06]	**-0.07,** **[-0.12, -0.01]**	**0.12,** **[0.07, 0.17]**	0.02, [-0.04, 0.08]
	61–80	-0.01, [-0.08, 0.06]	0, [-0.08, 0.08]	-0.07, [-0.15, 0.02]	0.06, [0, 0.12]	0.03, [-0.05, 0.1]
	>80	0.07, [0, 0.14]	0.01, [-0.07, 0.1]	**0.14,** **[0.05, 0.21]**	**-0.17,** **[-0.22, -0.12]**	-0.04, [-0.11, 0.03]
Education	<7	**0.12,** **[0.05, 0.19]**	-0.01, [-0.09, 0.08]	**0.19,** **[0.11, 0.28]**	-0.16, [-0.21, -0.12]	**-0.13,** **[-0.18, -0.07]**
	7–12	-0.04, [-0.11, 0.04]	0.07, [-0.03, 0.16]	**-0.12,** **[-0.21, -0.03]**	0.02, [-0.04, 0.09]	0.06, [-0.03, 0.15]
	>12	**-0.08,** **[-0.14, -0.02]**	-0.06, [-0.13, 0.03]	-0.07, [-0.14, 0.01]	**0.14,** **[0.08, 0.2]**	0.06, [-0.01, 0.15]
Sex (Proportion Male)		-0.05, [-0.12, 0.02]	0.02, [-0.07, 0.11]	**-0.08,** **[-0.17, -0.01]**	0.05, [0, 0.11]	0.05, [-0.02, 0.13]

Note. Significant contrasts at the .01 level indicated in bold. L = left, R = right, B = bilateral.

Estimated median difference of class members versus others and 99% credible intervals. Estimates for categorical variables reflect the difference in proportions between class members and others. Estimates for non-categorical variables reflect the difference in the value between class members and others. For lesion volume, the ratio between class members and others is reported.

**Table 3.** Characteristics of the 13-class solution.

		Class 1	Class 2	Class 3	Class 4	Class 5	Class 6	Class 7	Class 8	Class 9	Class 10	Class 11	Class 12	Class 13
Lesion Side	L	**.24,** **[.08, .38]**	.05, [-.08, .18]	**-.28,** **[-.39, -.11]**	-.01, [-.07, .06]	.12, [-.17, .38]	**.30,** **[.14, .43]**	.02, [-.1, .15]	**-.35,** **[-.42, -.24]**	**-.13,** **[-.23, -.02]**	**-.34,** **[-.41, -.24]**	-.09, [-.23, .06]	**.19,** **[.07, .3]**	**.27,** **[.14, .38]**
	R	**-.24,** **[-.37, -.08]**	-.08, [-.2, .06]	**.31,** **[.14, .44]**	-.01, [-.08, .07]	-.10, [-.35, .18]	**-.27,** **[-.39, -.12]**	-.04, [-.16, .08]	**.31,** **[.19, .40]**	**.12,** **[.01, .22]**	**.34,** **[.23, .43]**	.08, [-.07, .23]	**-.17,** **[-.27, -.06]**	**-.25,** **[-.35, -.13]**
	B	.0, [-.06, .11]	.03, [-.03, .12]	-.04, [-.08, .05]	.01, [-.03, .05]	-.03, [-.08, .19]	-.03, [-.07, .06]	.02, [-.04, .1]	.04, [-.03, .13]	.01, [-.04, .08]	0, [-.05, .07]	.01, [-.06, .11]	-.02, [-.06, .05]	-.03, [-.07, .05]
Lesion Volume		.83 [.13, 6.18]	.69, [.22, 2.56]	**6.32,** **[1.11, 31.35]**	**.37,** **[.2, .68]**	1.21, [.05, 28.9]	2.26, [.56, 9.22]	1.46, [.36, 6]	2.27, [.73, 6.64]	.91, [.34, 2.39]	2.22, [.87, 6.23]	.27, [.03, 2.84]	.98, [.38, 2.67]	1.65, [.47, 5.63]
Stroke Time (Acute)		.09, [-.06, .2]	.06, [-.06, .17]	-.04, [-.21, .1]	.03, [-.03, .09]	-.15, [-.42, .1]	.03, [-.12, .16]	.0, [-.12, .10]	-.02, [-.14, .10]	.06, [-.03, .15]	.02, [-.09, .12]	**-.14,** **[-.29, -.01]**	.07, [-.03, .16]	.01, [-.13, .12]
GCA		**.22,** **[.01, .44]**	**.30,** **[.19, .43]**	.0, [-.15, .15]	**-.26,** **[-.31, -.21]**	.33, [-.06, .67]	-.07, [-.2, .06]	**.38,** **[.23, .53]**	**.34,** **[.23, .46]**	0, [-.08, .10]	-.05, [-.13, .04]	**.36,** **[.17, .52]**	-.04, [-.13, .05]	**.16,** **[.01, .3]**
Fazekas		-.14, [-.66, .55]	**.31,** **[.02, .61]**	.17, [-.23, .53]	**-.25,** **[-.39, -.06]**	.28, [-.62, 1.37]	-.34, [-.67, .0]	.25, [-.11, .62]	**.37,** **[.06, .69]**	.01, [-.21, .28]	.12, [-.09, .35]	.32, [-.22, .89]	-.17, [-.38, .09]	.26, [-.06, .62]
Age (years)	18–60	-.07, [-.15, .06]	-.01, [-.09, .09]	-.09, [-.17, .03]	**.12,** **[.06, .17]**	.02, [-.14, .29]	.09, [-.04, .23]	-.09, [-.15, .0]	.05, [-.04, .15]	0, [-.08, .08]	.04, [-.05, .15]	-.03, [-.12, .09]	.02, [-.06, .11]	-.07, [-.14, .03]
	61–80	-.05, [-.2, .11]	-.04, [-.16, .09]	.09, [-.07, .26]	**.08,** **[.01, .14]**	-.11, [-.33, .17]	-.07, [-.2, .09]	-.03, [-.15, .09]	-.01, [-.12, .11]	.02, [-.08, .13]	.07, [-.04, .18]	.01, [-.13, .16]	.01, [-.09, .12]	.01, [-.12, .13]
	>80	.11, [-.04, .27]	.05, [-.07, .17]	.0, [-.15, .16]	**-.20,** **[-.25, -.14]**	.08, [-.16, .34]	-.02, [-.16, .12]	.12, [.0, .23]	-.04, [-.15, .08]	-.02, [-.11, .08]	**-.12,** **[-.21, -.01]**	.01, [-.11, .15]	-.04, [-.13, .06]	.06, [-.06, .19]
Education (years)	<7	**.18,** **[.02, .34]**	-.08, [-.17, .05]	.0, [-.14, .19]	**-.19,** **[-.23, -.14]**	.02, [-.17, .32]	-.11, [-.2, .04]	**.22,** **[.09, .34]**	-.06, [-.16, .06]	**-.15,** **[-.22, -.08]**	-.10, [-.18, .0]	**.20,** **[.06, .35]**	-.06, [-.14, .04]	.09, [-.04, .25]
	7–12	-.08, [-.24, .1]	-.04, [-.17, .09]	-.11, [-.29, .08]	.06, [-.01, .12]	-.21, [-.43, .08]	.13, [-.05, .29]	-.09, [-.21, .04]	.12, [-.01, .25]	**.18,** **[.08, .28]**	.05, [-.08, .17]	-.14, [-.28, .01]	**.14,** **[.03, .24]**	-.01, [-.16, .14]
	>12	-.10, [-.21, .04]	.11, [-.01, .25]	.10, [-.07, .29]	**.13,** **[.07, .19]**	.17, [-.08, .47]	-.03, [-.16, .14]	**-.13,** **[-.21, -.03]**	-.06, [-.17, .06]	-.03, [-.12, .07]	.05, [-.05, .17]	-.06, [-.17, .07]	-.09, [-.17, .01]	-.09, [-.19, .06]
Sex (Proportion Male)		-.07, [-.22, .10]	.02, [-.10, .14]	.01, [-.16, .17]	.07, [.0, .13]	.05, [-.23, .29]	.0, [-.15, .15]	-.09, [-.21, .02]	.11, [-.01, .23]	.01, [-.09, .11]	-.03, [-.14, .08]	-.02, [-.16, .13]	.01, [-.09, .11]	-.06, [-.19, .07]

Note. Significant contrasts at the .01 level indicated in bold. L = left, R = right, B = bilateral.

Estimated median difference of class members versus others and 99% credible intervals. Estimates for categorical variables reflect the difference in proportions between class members and others. Estimates for non-categorical variables reflect the difference in the value between class members and others. For lesion volume, the ratio between class members and others is reported.

### 3.3 Interpretation of the five profiles

**Class 1 (“Memory”, n** **=** **469)** was characterised by a comparably higher rate of numerical cognition and memory impairments coupled with an absence of visual field impairment and left egocentric neglect. Class 1 members were more likely left- than right-hemispheric stroke patients (E_LH_ = .54, 99% CI [.48, .61], E_RH_ = .40, 99% CI [.34, .47]) and had higher GCA ratings. Class 1 was associated with a single component of 233 network edges which were more disconnected relative to others. This component consisted of edges spread across all networks. Class 1 was not associated with any voxel clusters. Class 1 members were more likely to be lower educated.

**Class 2 (“Left Neglect”, n** **=** **288)** included primarily right hemisphere stroke patients (E_LH_ = .11, 99% CI [.06, .16], E_RH_ = .81, 99% CI [.73, .87]) with left egocentric and allocentric neglect. Class 2 members had comparably higher rates of visual field and executive function deficits. They were characterised by larger lesions and worse premorbid brain health. VLSM analysis identified two clusters of significant voxels associated with this class, impacting the right lateral occipital cortex, insular cortex, and angular gyrus (150 cm^3^, MNI = [46, -4, 2]) and the right middle frontal gyrus (0.13 cm^3^, MNI = [30, 14, 30]). Class 2 was associated with a network of 773 disconnections, the largest portion of which were within the right-hemispheric visual network (18% of all visual connections).

**Class 3 (“Language”, n** **=** **320)** was characterised by widespread impairment affecting language, numerical cognition, memory, and praxis. Class 3 was associated with larger lesions and left-hemisphere stroke (E_LH_ = .60, 99% CI [.52, .66], E_RH_ = .35, 99% CI [.29, .43]). Lesions in four voxel clusters in the left insula and putamen (16.3 cm^3^, MNI = [-32, 4, 8]), left precentral gyrus (0.35 cm^3^, MNI = [-46, 0, 42]), the left middle frontal gyrus (0.24 cm^3^, MNI = [-40, 24, 26]), and the left white matter (0.11 cm^3^, MNI = [-28, -60, 18]) were associated with Class 3. Class 3 was associated with a single network of 429 disconnections with the left-hemispheric default mode network most impacted (12% of all default mode connections). Class 3 members were also less likely younger and more likely lower educated.

**Class 4 (“No or mild impairment”, n** **=** **703)** was characterised by a low probability of impairment. Class 4 members had smaller lesions, better premorbid health, were more likely younger, and higher educated. Class 4 members were equally likely left- or right-hemispheric stroke patients (E_LH_ = .47, 99% CI [.42, .52], E_RH_ = .45, 99% CI [.40, .50]).

Finally, **Class 5 (“Attention”, n** **=** **392)** was characterised by non-lateralised cancellation task impairment accompanied by low probabilities of impairment across all other subtests. Class 5 members were more likely right-hemisphere stroke patients (E_LH_ = .28, 99% CI [.22, .35], E_RH_ = .64, 99% CI [.57, .71]) and less likely lower educated.

There was no significant voxel cluster or network-level disconnection pattern associated with Class 4 or Class 5.

### 3.4 Interpretation of the 13 profiles

#### 3.4.1 Left-lateralised profiles

**Class 1 (“Right-sided neglect”, n** **=** **91)** was characterised by right egocentric neglect and spared performance on the visual field test. In addition, Class 1 members exhibited a higher rate of sentence reading and numerical cognition impairment. Class 1 members were more likely left-hemispheric stroke patients (E_LH_ = .66, 99% CI [.50, .80], E_RH_ = .27, 99% CI [.14, .42]) with higher GCA ratings and a lower education level. There were insufficient lesion maps (n = 13) to investigate the association with lesion anatomy.

**Class 6 (“Global language & memory”, n** **=** **72)** was characterised by impairments on language (picture naming and sentence reading), verbal memory, and number writing. Class 6 had spared performance on the visual field test, and no left-sided egocentric neglect. Class 6 members were more likely left-hemispheric patients (E_LH_ = .71, 99% CI [.57, .84], E_RH_ = .24, 99% CI [.13, .38]). A cluster of significant voxels including the left insular cortex, putamen, and inferior frontal gyrus (pars opercularis) (volume = 28.8 cm^3^, peak z-score at MNI = [-30, 6, 12]), and a second cluster impacting the left insular cortex and left central opercular cortex (0.30 cm^3^, MNI = [-30, -20, 26]) was associated with Class 6. Class 6 was characterised by a component of 360 disconnections encompassing several networks, among which the left default mode network was most disconnected (12%).

**Class 12 (“Expressive language”, n** **=** **198)** was characterised by a low probability of impairment across all tasks, except for sentence reading, number writing, and picture naming, with mostly intact memory performance compared with class 6. Class 12 membership was associated with left-hemispheric stroke (E_LH_ = .62, 99% CI [.51, .72], E_RH_ = .33, 99% CI [.23, .43]) and two voxel clusters, one centred in the left Heschl’s Gyrus (0.19 cm^3^, MNI = [-42, -20, 8]) and the second within the left planum polare (0.18 cm^3^, MNI = [-44, -10, -6]). Class 12 was characterised by a single component of 186 disconnections, with the left-hemispheric default mode network being the most impacted (7%). Class 12 patients were more likely part of the middle education level than others.

**Class 13 (“Severe language & neglect”, n** **=** **107)** was characterised by high probabilities of impairment across language, numerical cognition, memory, praxis, visual field impairments, and right-sided neglect. Class 13 included mainly left-hemispheric stroke patients (E_LH_ = .69, 99% CI [.58, .80], E_RH_ = .26, 99% CI [.16, .37]) with higher GCA ratings. Class 13 was associated with 7 left-hemispheric voxel clusters: precentral gyrus (0.55 cm^3^, MNI = [-34, 2, 22]), inferior frontal gyrus (pars triangularis) (0.34 cm^3^, MNI = [-58, 30, 10]), insula (0.14 cm^3^, MNI = [-36, 6, -4]), hippocampus (0.12 cm^3^, MNI = [-36, -22, -12]), inferior frontal gyrus (pars opercularis) (0.10 cm^3^, MNI = [-58, 20, 24]), and two clusters in the white matter (0.96 cm^3^, MNI = [-40, -40, 2]; 0.88 cm^3^, MNI = [-40, -36, -6]). Class 13 was characterised by a component of 266 disconnections across many networks, among which the left dorsal attention network was most disconnected (8%).

#### 3.4.2 Right-lateralised profiles

**Class 3 (“Severe left-sided visuospatial impairment”, n** **=** **69)** mainly included right-hemispheric stroke patients (E_LH_ = .19, 99% CI [.08, .33], E_RH_ = .78, 99% CI [.62, .90]) who had impairments on tests involving a visual component. Class 3 was associated with larger lesions, and 9 significant voxel clusters mainly impacting the right visual cortex. The largest of these voxel clusters (70.14 cm^3^ volume) impacted the lateral occipital cortex (inferior division), middle temporal gyrus (posterior division), and the intracalcarine cortex (MNI = [24, -80, 6]). The remaining voxel clusters were centred in the right middle frontal/precentral gyri (0.86 cm^3^, MNI = [32, -6, 26]), amygdala (0.32 cm^3^, MNI = [20, -6, -10]), precentral gyrus (0.29 cm^3^, MNI = [56, 4, 32], supramarginal gyrus (0.29 cm^3^, MNI = [66, -36, 30]), inferior frontal gyrus pars opercularis (0.27 cm^3^, MNI = [60, 16, 6]), insular cortex (0.15 cm^3^, MNI = [32, -2, 14]), hippocampus (0.14 cm^3^, MNI = [30, -18, -16]), and putamen (0.13 cm^3^, MNI = [26, -4, 10]). Class 3 was associated with a component of 360 network-level disconnections with the right visual network being the most impacted (18%).

**Class 8 (“Severe left-sided neglect”, n** **=** **147)** was characterised by left egocentric and allocentric neglect, coupled with lower probabilities of impairment on picture naming and verbal recall tasks. Class 8 patients primarily had right hemisphere lesions (E_LH_ = .12, 99% CI [.06, .21], E_RH_ = .77, 99% CI [.67, .86]) and worse premorbid brain health. Class 8 was associated with a significant cluster of voxels in the right insular cortex and superior temporal gyrus (0.62 cm^3^, MNI = [48, -10, 0]). Class 8 was associated with a component of 476 disconnections among which the right visual network was most disconnected (12%).

**Class 9 (“Attention”, n** **=** **210)** was characterised by non-lateralised impairment on the cancellation test co-occurring with low impairment rates across other tests. The majority of patients had a right-hemispheric stroke (E_LH_ = .33, 99% CI [.24, .43], E_RH_ = .60, 99% CI [.49, .69]) and Class 9 members were less likely lower educated. Class 9 was not significantly associated with any voxels nor network-level disconnections.

**Class 10 (“Left-sided egocentric neglect”, n** **=** **151)** was characterised by left-sided egocentric neglect coupled with low impairment rates on other tasks mostly due to right-hemispheric stroke (E_LH_ = .13, 99% CI [.07, .22], E_RH_ = .80, 99% CI [.70, .88]). Class 10 was associated with significant voxel clusters in the right putamen, thalamus and white matter (3.88 cm^3^, MNI = [22, -18, 18]), right precentral gyrus (0.08 cm^3^, MNI = [44, -10, 32]), and postcentral gyrus (0.08 cm^3^, MNI = [44, -16, 32]). Class 10 members had a subnetwork of 493 disconnections among which the ventral attention network in the right hemisphere was most disconnected (11%).

#### 3.4.3 Non-lateralised profiles

Finally, there were five non-lateralised profiles (Fig. 6) which were not significantly associated with lesion side, location, and network disconnections.

**Class 2 (“Potential Premorbid cognitive impairment”, n** **=** **199)** was characterised by moderate impairment probabilities on memory tasks or the cancellation task. Notably, Class 2 members had worse premorbid brain health. Class 2 was not included in VLSM, as there was insufficient lesion overlap.

**Class 4 (“No or mild impairment”, n** **=** **656)** was characterised by a low probability of impairment across all subtests. Class 4 members had significantly smaller lesions, better premorbid brain health, more likely to be younger, and higher educated.

**Class 5 (“Mild right-sided neglect”, n** **=** **23)** was characterised by right-sided egocentric neglect. However, Class 5 consisted of few patients and, therefore, had a high uncertainty regarding the impairment probabilities and associated covariates.

**Class 7 (“Low cognitive reserve”, n** **=** **156)** was characterised by higher probabilities of impairment within the numerical and memory domains. Class 7 patients had higher GCA ratings and were more likely lower educated. There was insufficient lesion overlap to examine the relationship with lesion location.

**Class 11 (“Executive impairment”, n** **=** **93)** was characterised by executive function impairment which occurred in the absence of neglect and visual field impairments. Class 11 patients had higher GCA ratings, were less likely acute stroke patients, and more likely lower educated. There were not enough lesion maps (n = 9) available to assess Class 11’s relationship with lesion location.

### 3.5 Lesion neuroanatomy does not fully explain PSCI profiles

**Fig. 11.**
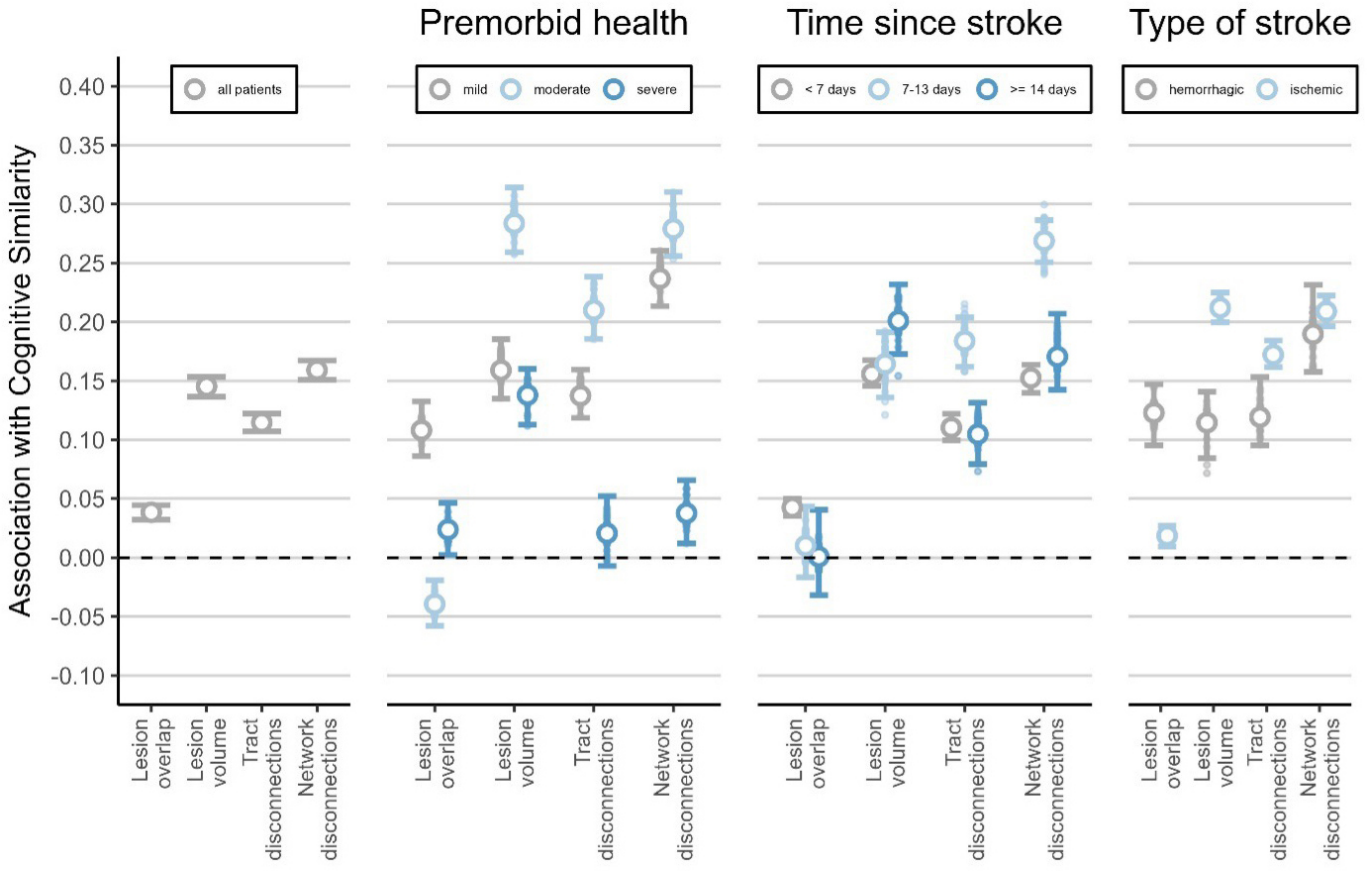
Associations of cognitive and neuro-anatomical similarity (lesion overlap, lesion volume, tract- and network disconnections). The error bars represent the 95% confidence intervals of the correlation. The small dots outside the error bars represent the extreme correlations based on a leave-one-out procedure to assess influential observations.

Last, we evaluated the extent to which the cognitive profiles as identified by the LCA model were driven by lesion neuroanatomy (Fig. 1). First, cognitive and neuroanatomical similarity was evaluated for all patients with a lesion map. Within this group, all lesion metrics were significantly positively associated with cognitive similarity (Fig. 11). Associations of network-level disconnectivity (r(514^1^) = .16, 95% CI [.15, .17]) and lesion volume (r(514) = .15, 95% CI [.14, .15]) were highest, followed by tract disconnections (r(514) = .11, 95% CI [.11, .12]) and lesion location (r(514) = .04, 95% CI [.03, .04]). However, these associations were small (<.20) (Gignac & Szodorai, 2016) indicating that the similarity in cognitive profiles was only partially explained by similarity in lesions and their corresponding disconnections.

Next, analyses were conducted to evaluate whether these results were modulated by premorbid brain health (i.e., Global Cortical Atrophy Score + Fazekas score), the type of stroke damage (e.g., haemorrhages vs. ischaemic stroke), and the time between stroke and testing. Within premorbid brain health analyses, the association between cognition and network-level disconnections was very low in the severe group (r(92) = .04, 95% CI [.01, .07]) and moderate in the two groups with less severe atrophy and white matter lesions (mild: r(94) = .24, 95% CI [.21, .26], moderate: r(89) = .28, 95% CI [.26, .31]) (Fig. 11). Lesion location had the highest association with cognitive profiles in the mild brain health group (mild: r(94) = .11, 95% CI [.08, .13], moderate: r(89) = -.04, 95% CI [-.06, -.02], severe: r(92) = .02, 95% CI [.00, .04]). Lesion volume had the highest association with cognitive profiles for the moderate brain health group (moderate: r(89) = .28, 95% CI [-.26, .31], mild: r(94) = .16, 95% CI [.14, .19], severe: r(92) = .14, 95% CI [.11, .16]). These results suggest a stronger association between lesion location (and corresponding tract and network-level disconnections) with the cognitive profile in patients with less severe atrophy and white matter lesions. In the group of patients with more severe atrophy and white matter lesions, lesion volume was the best predictor of cognitive profiles, suggesting that severity of impairments may become more important than the type of impairment.

For time since stroke, the association of cognitive similarity and disconnection profile was similar between the hyper-acute stroke patients (<7 days) (r(344) = .15, 95% CI [.14, .16]) and patients tested later after stroke (r(59) = .17, 95% CI [.14, .21]). The group tested in between 1- and 2-week post-stroke had the highest association (r(66) = 27, 95% CI [.25, .29]). As time after stroke increased, the association with lesion volume increased, progressing from .16 (95% CI [.15, .17]) to .20 (95% CI [.17, .23]), while the association with lesion location decreased from .04 (95% CI [.04, .05]) to .00 (95% CI [-.03, .04]).

Last, we assessed the associations between cognitive and neuroanatomical similarity in haemorrhagic versus ischaemic stroke patients. The disconnection profile had a similar association with cognition in both groups (haemorrhage: r(78) = .19, 95% CI [.16, .23], ischaemic: r(257) = .21, 95% CI [.20, .22]). Lesion volume was more strongly associated with cognition in the ischaemic (r(257) = .21, 95% CI [.20, .22]) than in the haemorrhagic group (r(78) = .11, 95% CI [.08, .14]), while lesion location was less strongly associated with cognition in the ischaemic (r(257) = .02, 95% CI [.00, .03]) than in the haemorrhagic group (r(78) = .12, 95% CI [.09, .15]).

## 4 Discussion

This study’s results indicate that patterns of PSCI can be captured by underlying behavioural classes, and that these classes cannot be entirely explained by differences in lesion anatomy. This study used data-driven analyses to identify viable class solutions (5-class and 13-class). While classes were differentially associated with broad anatomical characteristics, lesion anatomy alone was insufficient to explain class separation. Overall, these results provide novel insight into the underlying structure of PSCI impairment, providing important theoretical groundwork necessary to support future translational work and theoretical research.

### 4.1 Interpretation of cognitive profiles yielded by simple and complex class solutions

This investigation yielded two viable distinctions of PSCI profiles: a 5- and a 13-class solution. Some aspects of the simpler, 5-class solution are comparable with the PCA solutions reported by Corbetta et al. (2015) and Bisogno et al. (2021), but there are also key differences. That is, our 5-class solution captures two profiles of classic cognitive deficits which occur following left- and right-lateralised stroke (left stroke with aphasia, right stroke with neglect). However, our 5-class solution also captures profiles that do not represent such classical deficits. For example, class 1 included patients with non-language impairments (e.g., memory, numerical cognition) while class 5 captured right hemisphere (and some left hemisphere) patients with non-lateralised attention deficits without neglect. Importantly, our 5-class solution also reflects that not all stroke survivors exhibit severe stroke-related cognitive impairment (Class 4). This class was associated with smaller lesions, better premorbid health, younger age, and higher education levels, highlighting the protective role of brain and cognitive reserve (Casolla et al., 2019; Contador et al., 2023; Dacosta-Aguayo et al., 2014; Stebbins et al., 2008; Umarova, 2017). Importantly, this class cannot be conceptualised as representing patients without cognitive impairment as many patients in class 4 still exhibited cognitive impairment. Given the diversity of lesions and cognitive impairments in this class, considering a more detailed class structure provides more insight into different subtypes of comparatively mild PSCI.

The 13-class solution provided a richer perspective on PSCI, distinguishing 4 left-lateralised, 4 right-lateralised and 5 non-lateralised profiles. These non-lateralised profiles capture important variability in PSCI profiles, particularly with respect to premorbid cognitive status. In the complex solution, the right-lateralised profiles were characterised by different types of visual-attentional impairments. Specifically, class 3 captured severe and global neglect and visual field impairment which impacted performance on all tasks involving a visual component. This profile was linked to large lesions impacting regions within (and connections between) early visual areas and regions of the posterior parietal cortex traditionally associated with neglect (Moore, Milosevich et al., 2023). This captures the common clinical presentation of comorbid neglect and visual field impairment which can be difficult to behaviourally distinguish. A second group (class 8) exhibited left egocentric and allocentric neglect and was associated with lesions in the right insula and superior temporal gyrus (both regions associated with neglect; Chechlacz et al., 2012; Molenberghs et al., 2012; Moore, Milosevich et al., 2023). Interestingly, this group exhibited worse premorbid brain health compared with other groups. This result aligns with previous work suggesting that older patients with worse brain reserve were more likely to have spatial neglect (Umarova, 2017) and adds to this that patients with worse premorbid brain health have a higher risk of presenting multiple comorbid, rather than isolated neglect deficits.

The third right-hemisphere class (Class 10) captured cases of left egocentric neglect which occurred with few comorbidities, and was linked to lesions in the right putamen, thalamus, and pre- and postcentral gyri, which have previously been associated with directional motor biases and egocentric neglect (Grimsen et al., 2008; Sapir et al., 2007). Interestingly, this class was mainly linked to disconnection in the right ventral attention network, while the other neglect classes were mainly linked to visual network disconnection. Taken together, the three left-neglect classes align with previous conceptualisations of neglect as a deficit which represents a common symptom of multiple underlying causes (Husain et al., 2001; Mattingley et al., 1998). The last right-hemisphere class (class 9) included patients with non-lateralised attentional impairment. This class is analogous to class 5 from the 5-class model as it similarly includes patients who likely suffer from general attentional deficits.

In terms of left-hemisphere profiles, two classes captured patients with differing severity of aphasia. Class 6 represented the classical, pure aphasia profile (anomia, alexia, and agraphia) and was accordingly associated with large lesions affecting key language areas (Friederici, 2015; Oh et al., 2014; Viñas-Guasch & Wu, 2017). Class 13 included patients with aphasia occurring alongside widespread multi-domain impairments. In line with past work, this globally impaired group exhibited worse premorbid brain health (Casolla et al., 2019; Stebbins et al., 2008) and was associated with large lesions affecting language and memory regions (Lim & Alexander, 2009). Notably, these two profiles do not align with classic aphasia distinctions (Landrigan et al., 2021; Wilson & Hula, 2019). This finding suggests that subtypes which are often prioritised in the neuropsychological literature may capture theoretically important special cases rather than representing the symptom variability characteristic of the clinical population.

The third left-hemisphere group (Class 1) was characterised by right-lateralised neglect. We have previously found interhemispheric disconnections to be associated with right neglect (Moore et al., 2021), and the present study expands on this with interhemispheric disconnections mainly between left frontotemporal areas and right posterior parietal regions. The remaining group (Class 12) included patients with left hemisphere strokes and comparatively mild rates of language and numerical cognition impairment.

The remaining five cognitive profiles were not lateralised and were not associated with any significant lesion correlates but reflect the importance of premorbid brain health. First, Class 4 was characterised by low rates of cognitive impairments coupled with better premorbid brain health, younger age, and higher education levels (analogous to Class 4 from the 5-class solution). Class 2 was characterised by memory impairments and non-lateralised cancellation task impairment, likely capturing pre-morbid cognitive decline (Yanhong et al., 2013). Class 11 included patients with executive function impairment. Given that this class exhibited lower lesion sizes coupled with worse atrophy and white matter integrity, it is likely that executive dysfunction is more closely related to general brain health than to the acute stroke event. Class 5 captured a small portion of patients who exhibited right-lateralised neglect coupled with memory and praxis impairments. Finally, class 7 captured patients with numerical cognition and memory impairment who exhibited lower education levels and worse atrophy. These results highlight that the clinical picture of premorbid impairment and cognitive reserve can be distinctly and qualitatively different from classical PSCI profiles which are more linked to lateralised lesions.

### 4.2 Cognitive profiles cannot be fully explained by lesion anatomy

Although several profiles mapped onto lesion locations, lesion location itself played a limited role in explaining profiles, as lesion similarity was only weakly associated with cognitive similarity. Our results highlight key factors which limit the explanatory power of lesion location. Mainly, lesion location was less predictive of cognitive similarity for patients with worse premorbid health. This finding aligns with past work suggesting that the impact of stroke lesions may be modulated by general brain health (Hobden et al., 2024; Rost et al., 2022). For the mild brain health group, the stroke-induced disconnections were the best predictor of the cognitive profile. Lesion volume was less important, potentially reflecting patient’s ability to compensate for the impact of stroke as white matter tracts are more intact. In the moderate brain health group, stroke-induced disconnections and lesion volume were equally strong predictors of the cognitive profile. Lesion volume may play a more pronounced role for these patients, as they are less able to compensate for the stroke impact. Last, in the severe brain health group, where white matter tracts are severely impacted by white matter lesions, the stroke-induced functional disconnections have little predictive strength anymore. In this group, diffuse brain injury which has accumulated over time results in cognitive profiles that are no longer typical of focal strokes. The reduced impact of lesion volume for this group may also reflect the increasing role of cognitive impairment related to non-focal vascular changes in the brain.

Additionally, lesion anatomy was a better predictor of the PSCI profile in patients assessed in the hyper-acute phase (e.g., <7 days post-stroke). This non-linear relationship is likely driven by non-linear patterns of cognitive recovery occurring within the very early period post-stroke (e.g., steep initial recovery, followed by slower changes; Nijboer et al., 2013). This recovery dynamic make brain–behaviour relationships less clearly defined as time progresses following stroke (de Haan & Karnath, 2018; Karnath & Rennig, 2017). Additionally, some patients may receive targeted PSCI therapies. As therapy approaches (and individual response to treatment) differ dramatically, variance explained by anatomy may reduce as therapy time increases. Notably, disconnection patterns were identified as an important driver of cognitive variability as lesion-induced disconnections at the tract level and network level were stronger predictors of the PSCI profile than lesion location itself. This finding is in line with past studies illustrating that disconnection metrics help account for important variability in post-stroke brain–behaviour relationships (Griffis et al., 2021; Salvalaggio et al., 2020).

This study employed routinely collected data including a short cognitive screen and clinical neuroimaging. While this approach maximises the size and representativeness of this study, it is possible that this approach may not fully capture the association between lesion and cognitive profiles. For example, more extensive neuropsychological batteries could be used to tease apart more fine-grained relationships (Gell et al., 2024; Salvalaggio et al., 2020). This study also used data from three OCS language versions (Dutch, Italian, and English) which each has small differences in test materials and scoring procedures (e.g., age/education specific scoring). The analyses presented in Supplementary Materials S2 indicate that these test version differences have not significantly impacted the conclusions of this study.

In addition, in vivo tractography could be used to capture key sources of variability in disconnectivity which may account for a significant portion of the variance in cognitive profiles (Lim & Alexander, 2009; Rost et al., 2022). Additionally, the Fazekas ratings on the clinical CT scans are likely underestimating the importance of white matter integrity. While previous work has validated the Fazekas scale for use in CT (Rudilosso et al., 2017) and provided evidence that MR and CT imaging produce comparable results in lesion mapping analyses (Moore, Jenkinson et al., 2023), it is possible that the combination of MR and CT used in this study may have induced some variability into the neural results due to differences in sensitivity to lesion damage. However, past research has suggested that the benefits of combining MR and CT imaging in lesion mapping analyses generally outweigh this potential added noise (de Haan & Karnath, 2018; Moore, Jenkinson et al., 2023). This study used routine neuroimaging data collected within <31 days of stroke. Past studies have demonstrated that these data are of sufficient quality to detect established brain–behaviour relationships (Moore & Demeyere, 2022; Moore et al., 2024), but neuroimaging data collected in the early time window post-stroke (e.g., <1 day) may have comparatively low sensitivity to lesion damage (Lansberg et al., 2000; Mohr et al., 1995). Additionally, this study includes only scans demonstrating clearly visible lesion boundaries as determined by expert raters. This means that the high false negative rate associated with acute CT stroke imaging likely increased the proportion of patients excluded but does not necessarily reduce the utility of scans depicting clear lesion boundaries.

Importantly also, individual tests may not directly map onto a single cognitive function, in spite of their main source of variance mapping onto specific cognitive domains (Iosa et al., 2022; Moore et al., 2024). For example, individual OCS test scores may measure several (potentially dissociable) cognitive functions concurrently (Demeyere et al., 2015). Latent class models cannot distinguish between this case and true comorbidities, meaning that they can overestimate the number of subpopulations (Lubke & Muthén, 2005). This may lead to an overestimation of the number of true classes. Factor mixture models are theoretically the most plausible model to explain PSCI variances/covariances. Factor mixture models assume that there are dimensions (cognitive functions) underlying test performance, but that there are also distinct subpopulations (Lubke & Muthén, 2005). However, factor mixture models can only be used when data are available from several subtests loading onto the same cognitive function. This requirement is typically not met for large stroke datasets, meaning that more extensive behavioural data are needed before factor mixture modelling can be used to explore PSCI. Moreover, future studies must investigate the replicability of the PSCI profiles and investigate whether the PSCI profiles predict differential recovery (Demeyere & Moore, 2024).

Neuroimaging and neuroimaging-derived metrics were only available for a subset of the patients included in this study, and some behavioural classes (Classes 1, 2, 5, 7, and 11) had insufficient lesion data to facilitate statistical brain–behaviour inferences. While the identified clusters were largely consistent between the full sample and the subsample with available neuroimaging (Supplementary Materials S3), some profiles were less consistent due to the small number of patients with lesion maps (Class 5 and Class 11 in the 13-class model) and could, therefore, not be included in the VLSM and network analysis. Future work with access to larger imaging samples is needed to clarify the neural correlates of these classes. Notably, even though lesion data were not available for all patients, the subsample included in these analyses is substantially larger than previous similar studies. For example, Filler et al. (2024) reported average sample sizes of 397 and 218 for studies investigating the relationship between PSCI and white matter hyperintensities and atrophy, respectively. Previous work has shown that lesion mapping analyses (both univariate and multivariate) are prone to some degree of results mislocalisation (Ivanova et al., 2021; Mah et al., 2014). For this reason, this study does not aim to draw causal associations between brain–behaviour relationships but instead aims to provide a qualitative description of the lesion profiles associated with each identified behavioural profile. Future studies can also aim to explore the extent to which specific neuroimaging analysis parameters (e.g., normalisation algorithm, normalisation template) may influence the results of lesion mapping analyses.

Overall, the results of this study reveal that PSCI is heterogeneous, encompassing both domain-specific profiles linked to focal lesions sites and profiles that are more strongly associated with premorbid health and demographic factors. Focusing merely on low-dimensional solutions of PSCI may reveal the strongest factors, but more complex solutions may help capture critical cognitive profiles which more accurately capture the variability present in real-world clinical populations. Future clinical studies can aim to build on this work by exploring whether cognitive profiles can be used to inform clinical care by evaluating associations with both cognitive, physical, and quality of life recovery outcomes.

## Data and Code Availability

The behavioural data of the UK-OCS are publicly available on the DPUK platform. The OCS-NL data and main scripts to replicate our analysis are available on https://doi.org/10.6084/m9.figshare.28387994. The lesion masks can be made available upon request.

## Supplementary Material

Supplementary Material

## Footnotes

1 The number of patients on which the association was based.
