# Supplementary material for: Post-stroke cognitive impairment: More than a lesion-symptom model
Source: Imaging Neurosci (Camb). 2025 Dec 11;3:IMAG.a.1050. doi: 10.1162/IMAG.a.1050 (PMC13288497; doi:10.1162/IMAG.a.1050)
Supplement: Supplementary Material [file IMAG.a.1050_supp.pdf]

Supplementary Materials 1. Description dataset

Patient recruitment

Patient data was collated from several previously published datasets (Table S1.1). Each study used slightly different selection criteria (Table S1.1). Patients were recruited in several studies with various selection criteria from study to study (Table S1.1). In the UK studies patients were either recruited within 3 weeks or 10 weeks post-stroke. In Italy, patients were included from 72 hours until 6 months post-stroke. In contrast, in Belgium there were no explicit criteria regarding time post-stroke. All studies recruited adult stroke patients. In the Italian study, only first-ever stroke patients were recruited, while in the UK and Belgian studies this was not a requirement to participate. Studies recruited patients at acute stroke units and rehabilitation centers.

Table S1.1. Information about patient recruitment

Country	Reference	N	Hospital	Time since stroke	Age range (years)	Study-specific In- and exclusion criteria
UK	(Demeyere et al., 2015)	208	Acute stroke unit Oxford	≤ 3 weeks	>=18 (no maximum)	/
UK	(Demeyere et al., 2016)	200	Acute stroke unit Oxford	≤ 3 weeks	Not reported	/
UK	(Demeyere et al., 2019)	821	37 sites	≤ 10 weeks	18 - 90	Able to concentrate for 1 hour.<br>Have sufficient language comprehension to pass first OCS tests.
Italy	(Iosa et al., 2022b; Mancuso et al., 2018)	684	14 rehabilitation centers	≥72 hours to ≤ 6 months	18 - 90	First-ever stroke.<br>No premorbid psychiatric or neurological disease.
Belgium	(Huygelier et al., 2022)	197	3 acute stroke, 3 rehabilitation units	≤ 6 months	>=18 (no maximum)	/
Belgium	(Thielen et al., 2024)	85	1 acute stroke, 2 rehabilitation units	No limit	>=18 (no maximum)	Able to consent<br>No ADHD, ASS, Schizophrenia

Behavioural data: missing data

A total of 577 patients did not complete all OCS subtests. For these patients, the average number of missing subtests was 2.5 (Mdn = 1, SD = 2.6). Only 60 participants had missing data on more than 6 of the 12 subtests (Figure S1.1A). The Little MCAR test demonstrated that missing data was not completely random (*MCAR(2525) = 5100, p < .01, 226 missing patterns*). Missing data occurred more frequently for certain subtests, such as the executive function and cancellation test (Figure S1.1B).

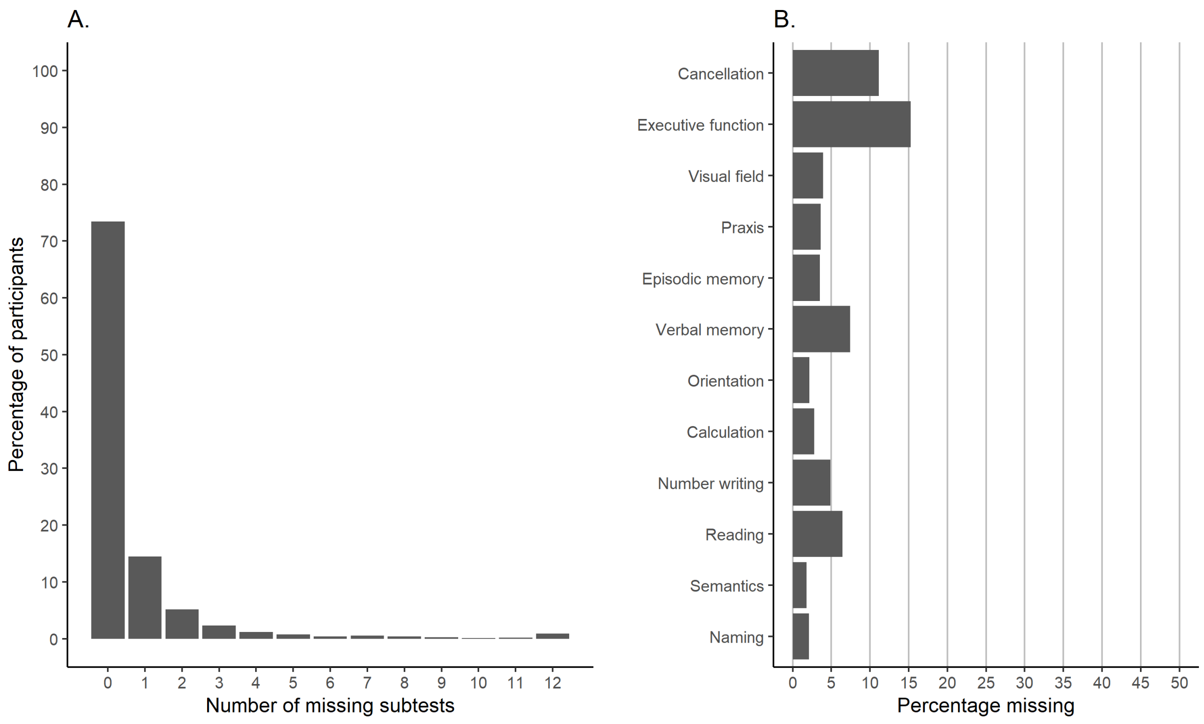

Figure S1.1. Distribution of the number of missing subtests (A) and the percentage missing observations per OCS subtest (B).

There was evidence for a significant difference between patients who completed and did not complete the OCS in age and years of formal education (Table S1.2). Completion rates depended on country with 90% of patients completing all subtests in the Belgian dataset, while this was 71% for the UK and 70% for the Italian dataset. In addition, patients with ischemic stroke were more likely to complete the OCS (76.5%) than patients with a haemorrhagic stroke (69%). According to the Bayes Factor (BF), there was no association of OCS completion with lesion side and the relation with sex was unclear (Table S1.2).

Table S1.2. Patient characteristics associated with incomplete OCS data.

	Complete OCS	Incomplete OCS	Statistical test	Result
Age (years)	M = 70, Mdn = 72, SD = 13.5	M = 73.6, Mdn = 77, SD = 13.7	Welch two-sample t-test	t(912.92) = -5.21, p < .001, 95% CI = [-4.9, -2.2], BF_{10} = 44614
Education (years)	M = 10.91 Mdn = 11 SD = 3.9	M = 9.96 Mdn = 10 SD = 4.1		t(637.83) = 4.19, p < .001, 95% CI [0.51, 1.40], BF_{10} = 548.4
Country (UK, IT, BE)	n = (859, 482, 254)	n = (347, 202, 28)	Bayesian Contingency Table Test	BF_{10} > 5000
Type of stroke (Ischemic, Haemorrhage, unknown)	n = (1076, 232, 287)	n = (331, 104, 142)		BF_{10} = 68
Lesion side (Left, Right, Bilateral)	n = (531, 661, 93)	n = (219, 220, 26)		BF_{10} = 0.06
Sex (Female, Male)	n = (691, 902)	n = (273, 292)		BF_{10} = 0.55

Supplementary Materials 2. Behavioural analysis

Model comparison

The LCA models were fit with the poLCA R package (Linzer & Lewis, 2011).

To determine the optimal number of classes, different LCA models are contrasted with increasing number of classes until the model no longer converges. For each contrast, the difference in the AIC and BIC indices was computed (Table S2.1). In addition, the Likelihood Ratio Test was performed (Table S2.1). The AIC index and Likelihood Ratio Test indicated a 13-class model as the best fitting model, while the BIC indicated the 5-class model as the best fitting model. The relative entropy for each number of classes decreases from a model with two classes to a model with five classes and remained relatively stable after this point (Figure S2.1). The probability that a participant belongs to a class for the 5- and 13-class solution are depicted in Figure S2.2, for the class to which the participant was assigned to and the other classes.

Table S2.1. Model comparison

M0 (k)	M1 (k+1)	Δ AIC	Δ BIC	LMR (17)	P-value
1	2	-3532.94	-3436.32	3412.53	< .001
2	3	-678.17	-581.55	681.34	< .001
3	4	-301.37	-204.76	320.86	< .001
4	5	-107.25	-10.64	135.14	< .001
**5**	6	-70.28	26.34	99.76	< .001
6	7	-52.17	44.45	82.44	< .001
7	8	-34.67	61.95	65.70	< .001
8	9	-22.48	74.14	54.04	< .001
9	10	-14.08	82.54	46.00	< .001
10	11	-10.67	85.95	42.73	< .001
11	12	-12.32	84.30	44.31	< .001
12	13	-6.24	90.38	38.50	< .001
**13**	14	7.83	104.45	25.03	.09

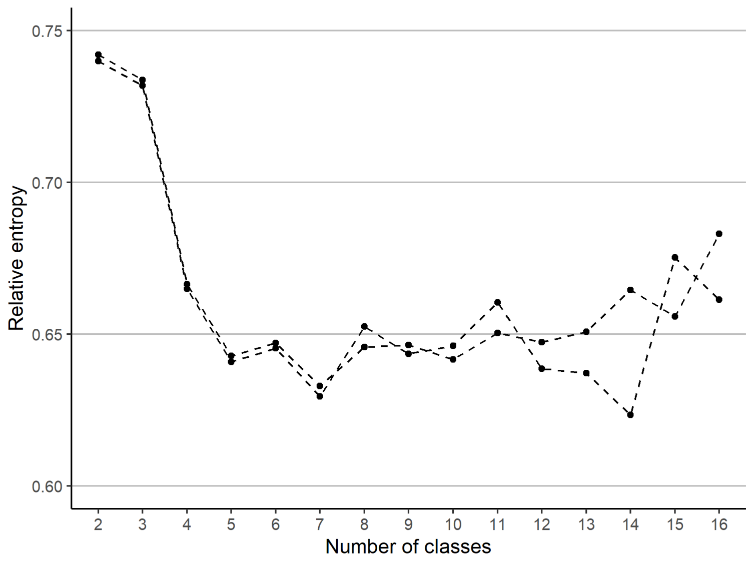

Figure S2.1. Relative entropy for each estimated model. The entropy for all cases and for all cases except 8 patients with imputed age are shown.

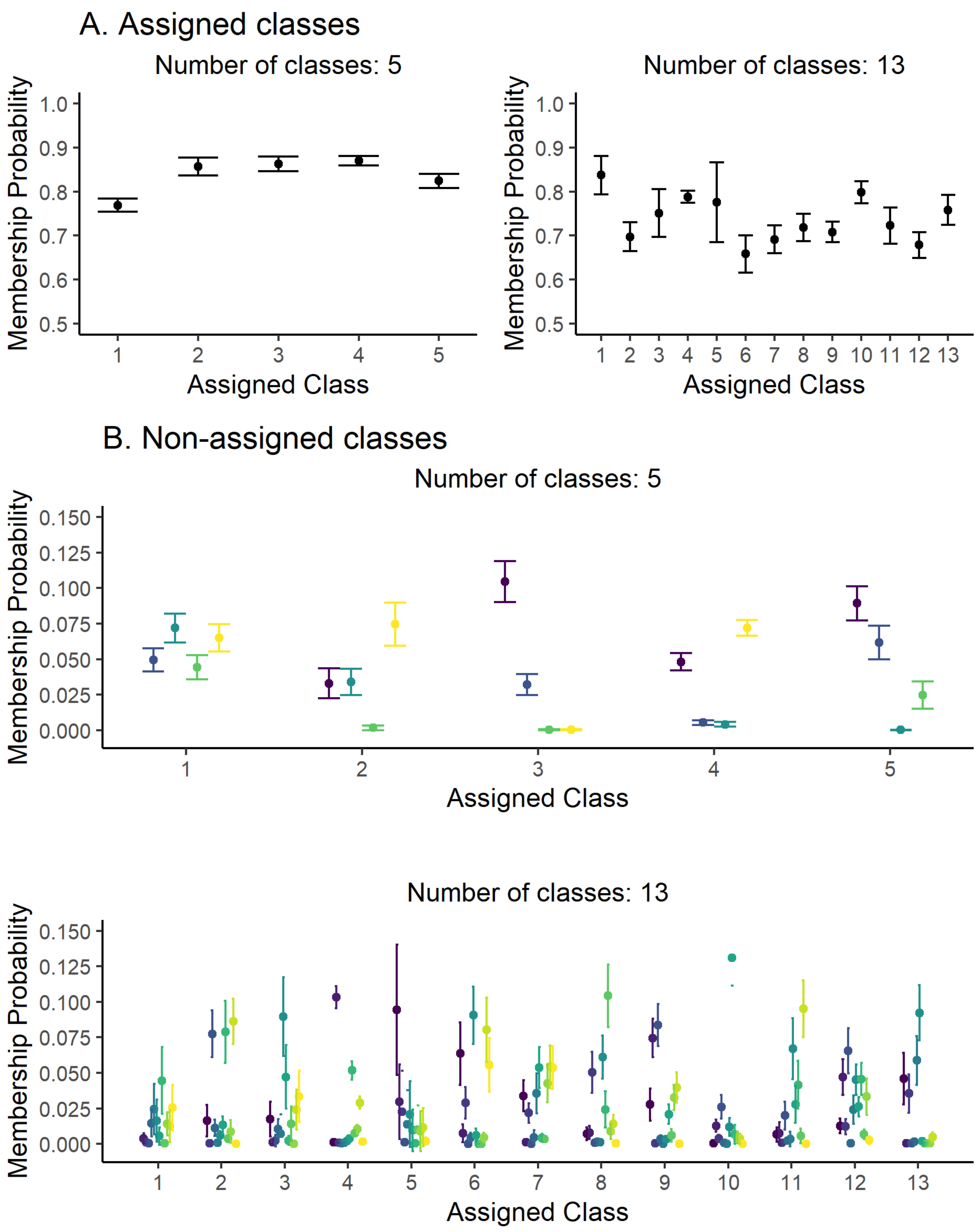

Figure S2.2. Membership probabilities for each class (separate error bars) for the assigned classes (A) and for the non-assigned classes (B) for the 5-class and 13-class solution.

Additional information 13-class solution

Classes differed in the number of impaired OCS subtests (Table S2.2). The probability of impairment on each OCS subtest for each class is visualized in Figure S2.3. For each subtest the classes are ordered according to the probability of an impaired performance on the subtest. These results reveal that each task contributed to the classification, as none of the tasks are equivalent in impairment probability between the 13 classes.

Table S2.2. Expected number of impaired subtests for each class

Class	M	95% CI	
4	0.61	0.55	0.67
2	2.30	2.06	2.55
9	2.88	2.65	3.12
10	3.89	3.61	4.19
12	4.00	3.73	4.28
11	4.52	4.13	4.92
8	6.04	5.69	6.39
1	7.01	6.52	7.50
5	7.30	6.33	8.26
6	7.78	7.24	8.31
7	8.06	7.69	8.43
3	8.98	8.39	9.56
13	11.58	11.19	11.98

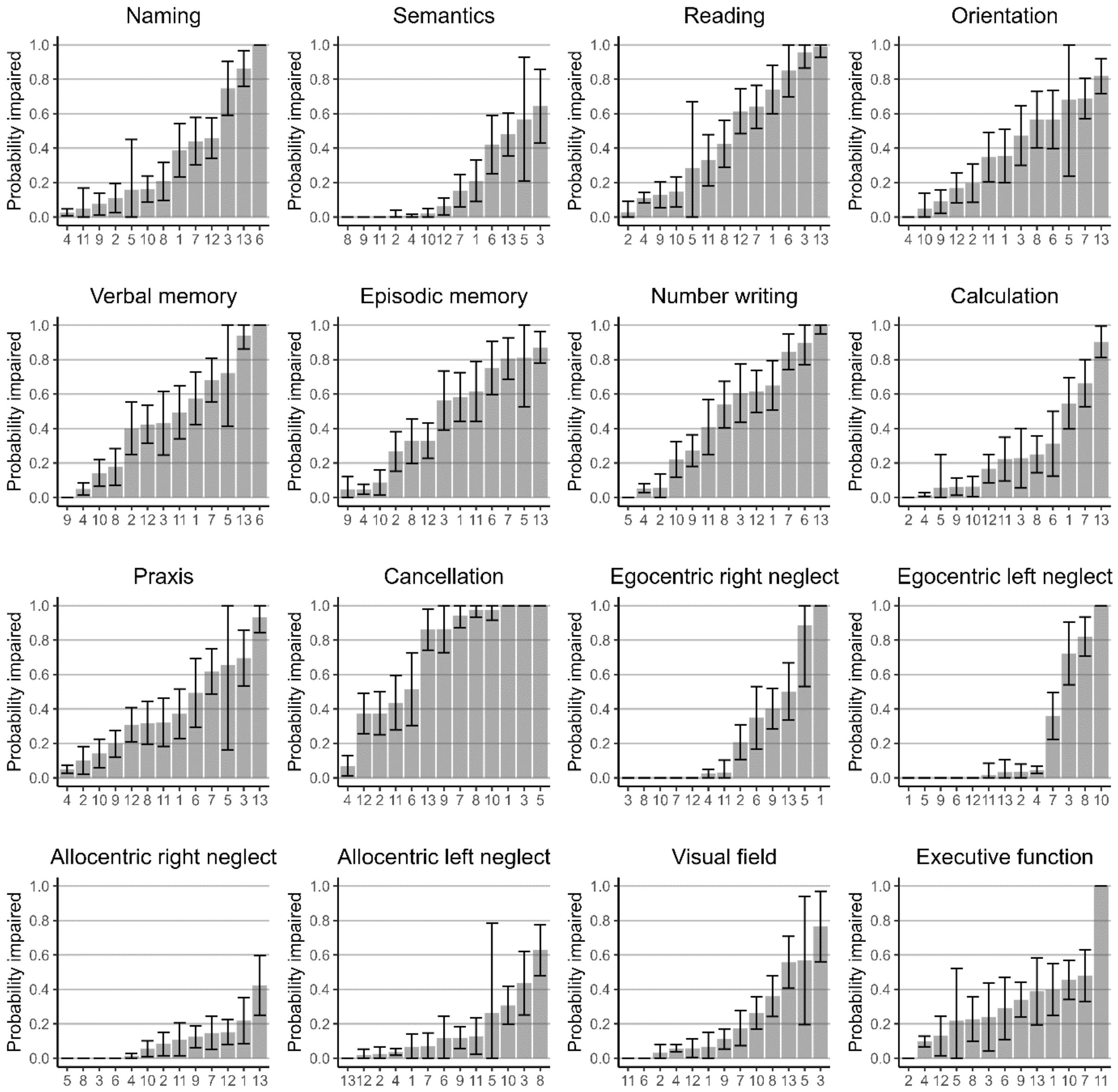

Figure S2.3. Probability of impairment per class on each OCS subtest sorted in ascending order per panel. The error bar represents the 95% confidence interval.

Demographic characteristics of classes

Method of analysis

To account for the uncertainty of the class memberships (Bakk & Kuha, 2021), we estimated the probability of observing each demographic covariate level (i.e., age, education, sex, time since stroke and lesion side) given a certain latent class using a Bayesian multinomial logistic regression model that considered the measurement error of the latent class predictor (Elliott et al., 2020). The Bayesian measurement error regression model was implemented in Stan (Stan Development Team, n.d.) and fitted with RStan (Stan Development Team, 2020). Then, based on the estimates, the expected difference and their corresponding 99% credible intervals in proportions per covariate level were computed between members of the class versus all others.

For lesion volume a linear regression model was used with lesion volumes log-transformed to correct for their skewness. For the GCA and Fazekas, hierarchical ordinal regression models were used, treating the individual scale items as repeated within-subject measures. The models for lesion volume, GCA and Fazekas ratings were fit using the R package brms (Bürkner, 2017) with default priors, 4 chains and 2000 iterations per chain. All regression models converged with Rhat values smaller than 1.05 and good mixing of traces. The model fit was evaluated through graphical posterior checks with the R BayesPlot package (Gabry & Mahr, 2024).

Since a complete cases analysis is associated with a high risk of bias when missing data is not at random (Buuren & Groothuis-Oudshoorn, 2011), we used multivariate imputation by chained equations to impute missing data using information from all available variables in the dataset (Buuren & Groothuis-Oudshoorn, 2011). Predictors to impute missing covariates were determined based on the proportion of usable cases (min. 20%) and the spearman rho correlation (> .10 or < -.10) (Buuren & Groothuis-Oudshoorn, 2011). The resulting predictor matrix is visualized in Figure S2.4. The predictive mean matching method was used to impute missing observations for all subtest scores, age, years of formal education and days since stroke (Buuren & Groothuis-Oudshoorn, 2011). For all categorical variables, a logistic regression or multinomial logistic regression model was used depending on the number of categories (Buuren & Groothuis-Oudshoorn, 2011). The convergence of the imputation model

was visually checked using trace plots. All chains mixed, thus showing good convergence. In addition, distributions of imputed and observed values were visually inspected for impossible values resulting from the imputation.

We created 5 imputed datasets and performed the analysis assessing the relationship between demographic variables and LCA clusters on each imputed dataset and the original complete-cases dataset. The estimates were highly similar between all datasets (Figure S2.5).

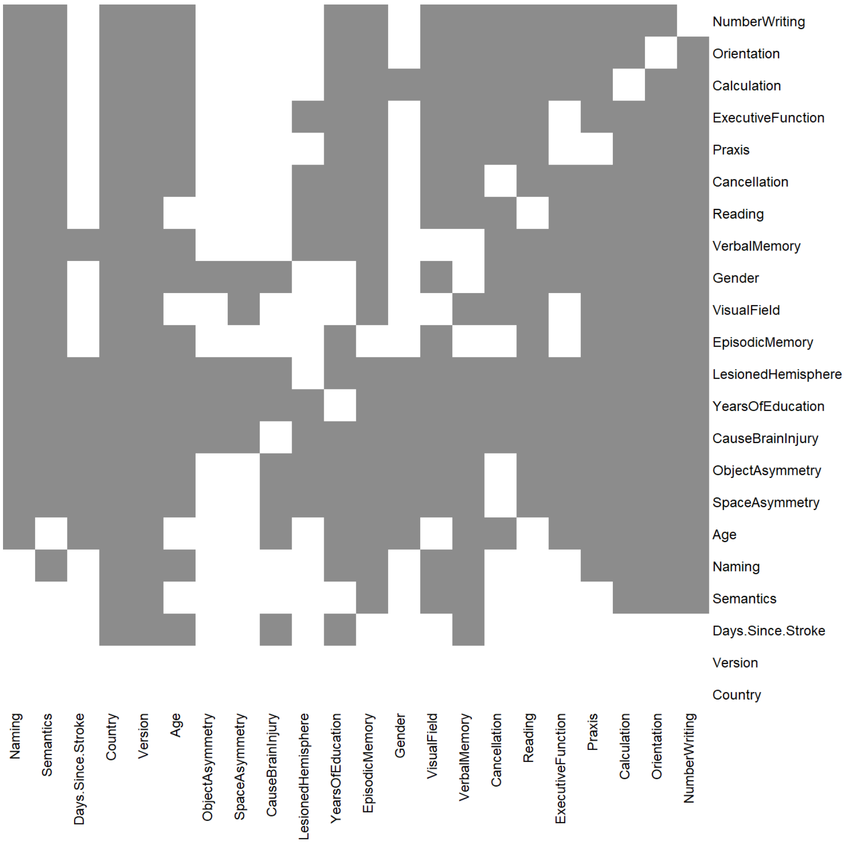

Figure S2.4. The predictor matrix. A grey square indicates that the variable in the column is used to predict the variable in the row.

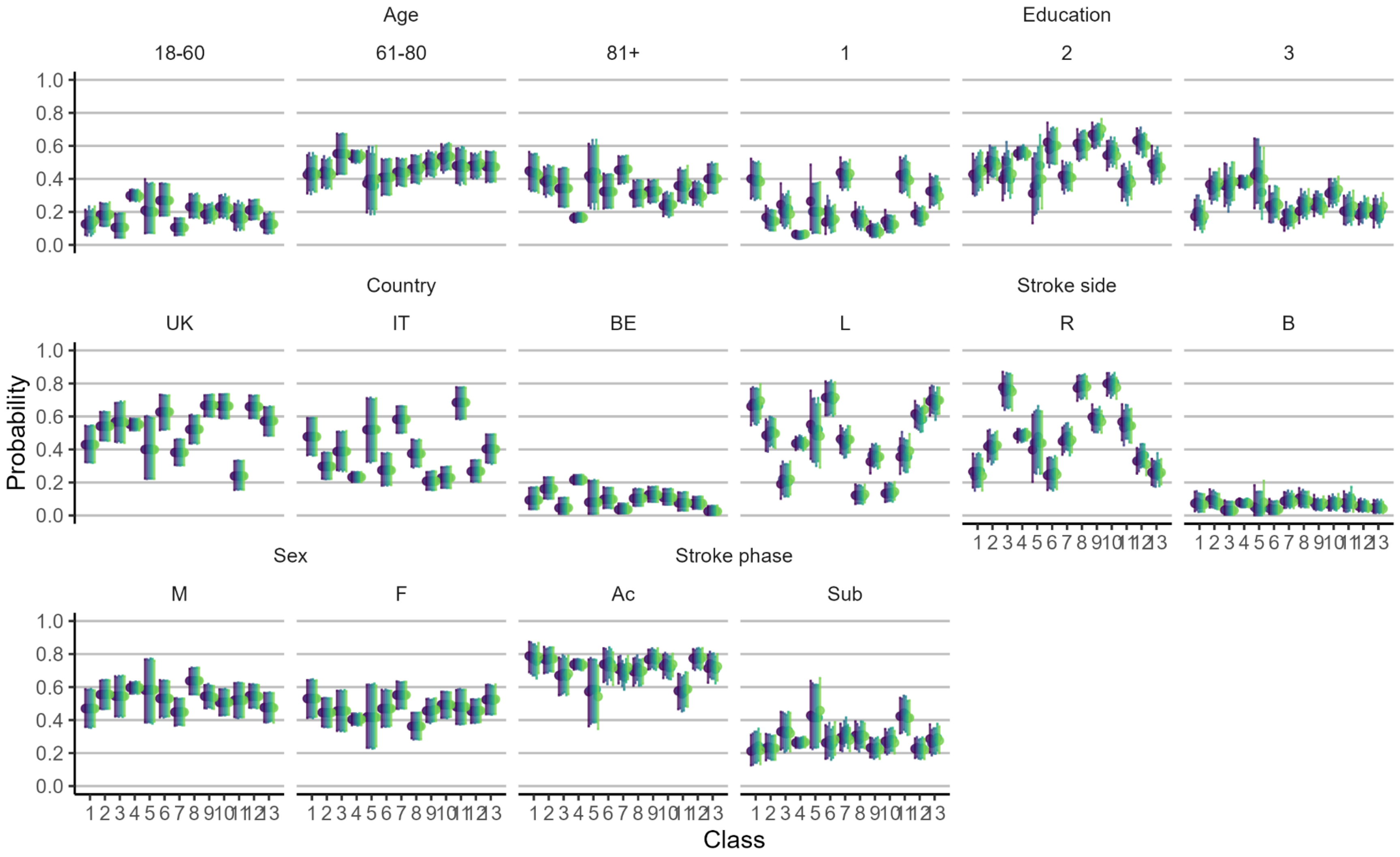

Figure S2.5. Point estimates and 95% credible intervals for the probability of observing a specific outcome given the latent class for the dataset with complete cases and 5 imputed datasets (different coloured bars).

Table S2.3. Participant characteristics for each assigned class.

		**Age**					**Days since stroke**					**Education**					**Country (%)**			**Stroke side (%)**			**Sex (%)**	
Class	*n*	*M*	*Mdn*	*SD*	*Range*		*M*	*Mdn*	*SD*	*Range*		*M*	*Mdn*	*SD*	*Range*		*UK*	*IT*	*BE*	*L*	*R*	*B*	*F*	*M*
1	72	75.53	79.00	11.83	31	92	19.26	8.00	33.96	1	205	8.52	9.00	3.82	3	20	43	49	08	68	26	6	53	47
2	129	74.23	77.00	13.02	26	92	16.11	5.00	29.98	1	218	11.13	11.00	4.01	3	23	56	29	16	49	42	9	44	56
3	64	73.45	75.50	13.45	18	95	22.19	7.50	31.55	0	125	10.37	11.00	4.09	3	18	58	39	03	19	79	2	45	55
4	775	67.15	69.00	13.18	24	96	16.47	7.00	27.06	0	423	12.04	12.00	3.76	3	30	55	23	22	44	48	08	40	60
5	22	72.50	72.50	12.09	46	90	38.16	10.00	71.25	1	293	10.50	9.50	4.60	5	18	41	55	5	59	41	0	41	59
6	78	70.41	72.50	15.44	31	94	28.24	8.00	58.85	1	323	10.80	10.50	3.66	5	23	63	28	9	72	25	3	46	54
7	149	76.85	79.00	12.23	24	97	23.90	11.00	40.28	0	285	8.17	8.00	4.27	1	25	39	58	3	47	44	9	55	45
8	137	71.11	73.00	14.17	21	93	20.71	6.00	34.53	0	235	10.09	10.00	3.47	3	23	53	37	9	12	78	10	36	64

9	197	72.28	73.00	12.65	23	93	17.74	7.00	30.93	0	273	10.59	10.00	3.11	4	20	66	22	12	33	60	7	46	54
10	158	70.72	72.00	12.58	34	96	23.79	9.00	58.41	0	567	10.94	11.00	3.71	3	20	66	23	11	13	81	6	49	51
11	85	74.41	77.00	11.77	29	93	24.44	14.00	32.22	0	199	8.35	8.00	3.92	3	23	25	69	6	36	58	7	48	52
12	189	71.60	74.00	13.25	25	94	14.12	5.00	21.89	0	181	9.83	10.00	3.41	2	20	66	28	7	61	34	5	45	55
13	117	75.95	78.00	11.87	40	98	27.80	11.00	45.51	1	235	8.84	9.00	3.75	2	18	58	40	2	70	26	4	53	47

Relation between 5- and 13-class solution

We examined how the two class solutions were related to each other (Figure 2.6). Some classes from solution 13 had strong associations with solution 5. That is, 70% of the class 1 members of the 13-class solution were members of class 1 in the 5-class solution. 90% of class 4 members were also members of class 4 in the 5-class solution. 91% of class 8 members were members of class 2 in the 5-class solution. 76% of class 9 were members of class 5 in the 5-class solution. And 88% of class 11 and class 12 were members of class 1 in the 5-class solution. Last, all members of class 13 were members of class 3 in the 5-class solution. Other classes of the 13-class solution had a weaker link to the classes in the 5-class solution, as they consist of mixes of at least 2 classes of the 5-class solution.

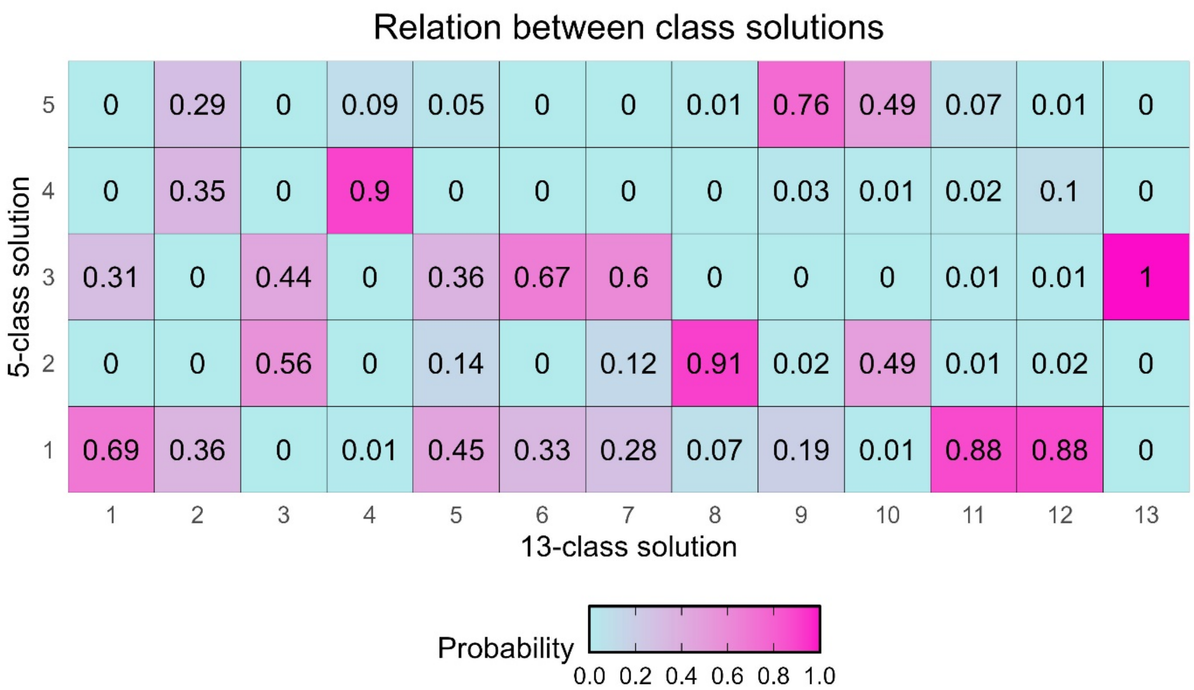

Figure S2.6. The probability that a patient belongs to a class of the 5-class solution given their membership in the 13-class solution.

Supplementary Materials 3. Neural data-analysis

Association of cognitive profiles and lesion side

The lesion side was derived from radiologist reports and was available for 1750 (81%) patients. Missing data were imputed according to the procedure described above and several imputed datasets were analysed to assess the impact of imputation (Figure S2.6). The association between behavioural classes and lesion side was examined with the same statistical approach as used for the demographic covariates of class members.

Association of cognitive profiles and lesion neuro-anatomy

We first assessed how many patients had a lesion map available per cognitive profile for the 5- and 13- class model and the stability of the cognitive profiles across the total set and subset of patients with a lesion map. The proportion of patients with an impairment are similar between both subsets for each class of the 5-class model (Figure S3.1). For the 13-class model, the majority of profiles are also similar between the patients with and without a lesion map (Figure S3.2), except for Class 5 and Class 11. Class 5 and Class 11 consist of less than 10 patients with a lesion map and are therefore excluded from the VLSM and network analysis (Table S*).

Table S*. Availability of lesion maps per class and model

	Patients with lesion map	Total patients
	**5-class solution**	
Class 1	103	467
Class 2	75	267
Class 3	72	318
Class 4	167	773
Class 5	99	347
	**13-class solution**	
Class 1	13	72
Class 2	32	129
Class 3	17	64
Class 4	167	775
Class 5	4	22
Class 6	25	78
Class 7	22	149
Class 8	37	137
Class 9	50	197
Class 10	57	158
Class 11	9	85

Class 12	52	189
Class 13	31	117

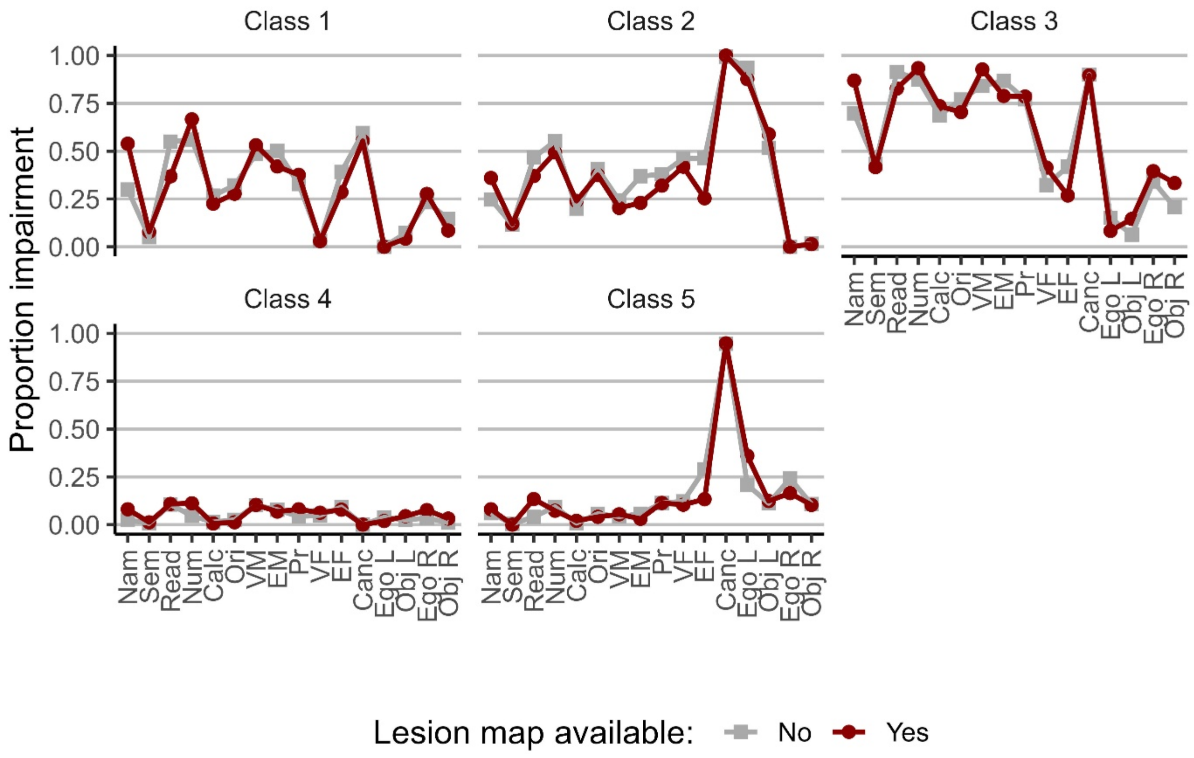

Figure S3.1. Cognitive profiles of for patients with versus without a lesion map for the 5-class model.

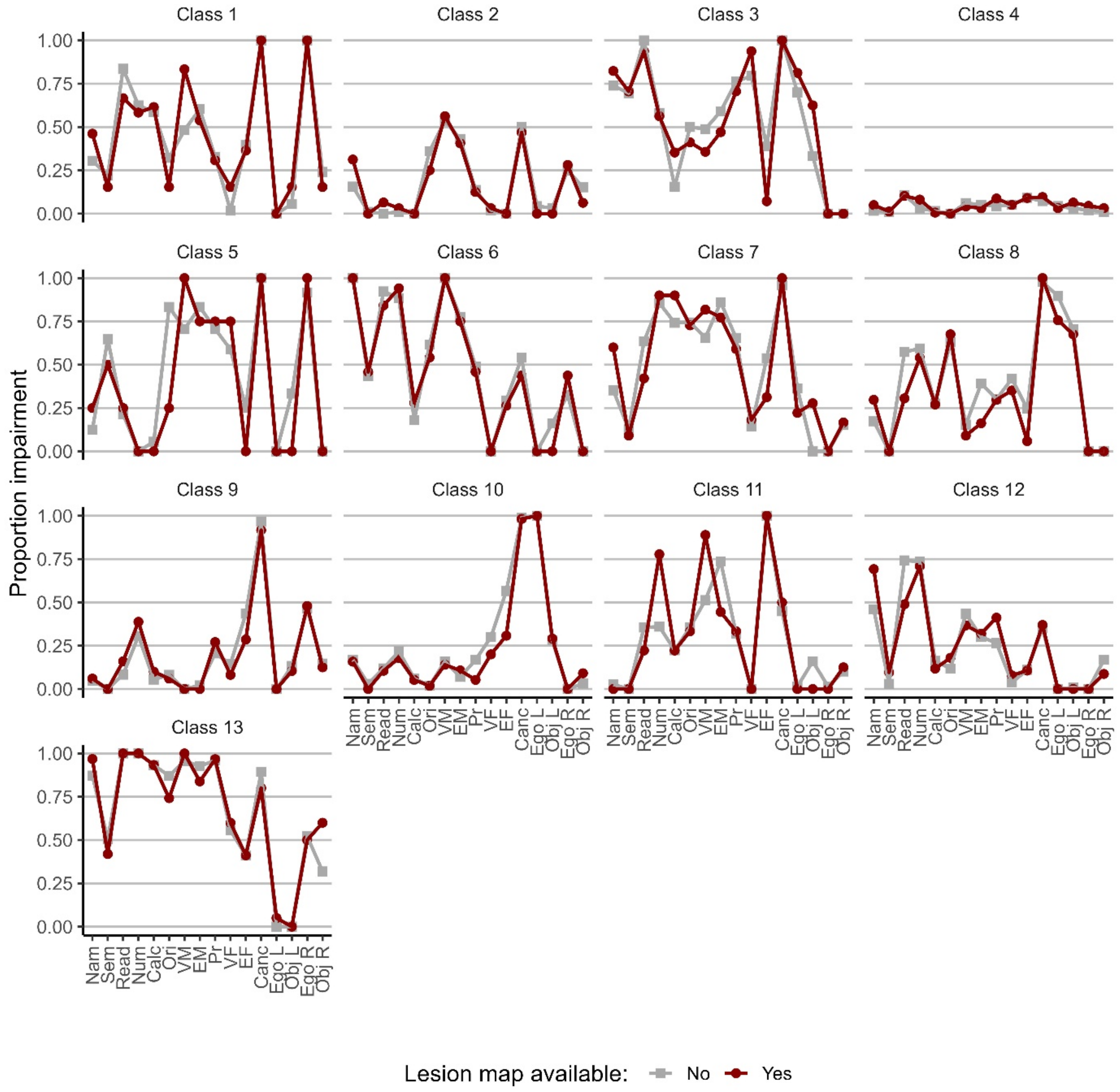

Figure S3.2. Cognitive profiles for patients with versus without a lesion map for the 13-class model.

VLSM Analysis Method

To assess the association of classes and lesion location, a one-sided Liebermeister test was performed in NiiStat (i.e., testing the hypothesis that a lesion in a voxel increases the probability of belonging to a certain class), controlling for lesion volume as a covariate. Voxels damaged in less than 10 patients were excluded from analyses.

Power and sensitivity analysis

We conducted a sensitivity analysis to determine which effect sizes we could identify with at least 80% power given our sample size. This information provides insight in the sensitivity of our VLSM analysis.

Method

We first determined the threshold for a voxel to be considered statistically significantly associated to behavioural impairment at a family-wise error rate of 5% using NiiStat. We therefore performed a VLSM including all voxels in the analysis in which at least 5 patients had a lesion and using permutation correction with 2000 permutations with lesion volume as a covariate. This analysis resulted in 141,922 voxels to be included in the VLSM analysis and a critical z-score ranging from -4.49 to -4.72 depending on the behavioural class. We then determined the alpha level corresponding to a statistical test of a single voxel based on the minimum and maximum critical z-scores (alpha: 0.000002 and 0.000004). A single voxel is considered statistically significantly associated with behavioural impairment when the p-value is lower than this alpha level.

We then simulated contingency tables based on four parameters. (1) The total number of patients (N). This parameter was fixed to our sample size (n = 515). (2) The number of patients who have a behavioural impairment (k) (i.e., belong to a specific behavioural class). The simulated range of k was based on the sizes of the behavioural classes for which lesion maps were available and ranged from 5 to 50. (3) The number of patients who have a lesion in a voxel (n). The range of n was based on the maximum lesion overlap in our sample (n = 80) and the minimum lesion overlap for voxels to be included in the VLSM (n = 5). (4) The last parameter was the number of patients who have a lesion in the voxel and a behavioural impairment (q). Q ranged from 1 to a maximum equal to all patients with a behavioural impairment or all patients with a lesion in a voxel. Moreover, only contingency tables where the odds ratio was above or equal to 1 were simulated, as this represents a higher probability of behavioural impairment when a patient has a lesion (i.e., corresponding to the one-sided hypothesis under examination).

A total of 1000 contingency tables were simulated for each set of parameters. For each simulated contingency table, the p-value was calculated. We used the one-sided

Liebermeister test to determine the p-value for the relation between behavioural impairment (binary) and lesion in a voxel (binary). The p-value was directly inferred from the hypergeometric distribution using four parameters (i.e., n+1, (N-n)+1, k+1 and q+1). Then, the proportion of significant p-values across the 1000 simulated tables was computed (i.e., power).

Results

The results reveal that the number of patients with a behavioural impairment and a lesion in the voxel affect the sensitivity of the VLSM analysis (Table S3.2, Figure S3.2). The highest sensitivity (lowest detectable odds ratio) is achieved when both or equal to 50 (Table S3.2). Moreover, the simulation revealed that for rare behavioural impairments (k=5), we only reach 80% power if 80% to 100% of the patients with the behavioural impairment also show a lesion in the voxel (Figure S3.3). The latter corresponds to very large odds ratios (OR>1000, Table S3.2). Similarly, for voxels that are only lesioned in 5 patients, 80% power could not always be obtained (Figure S3.2, Table S3.2).

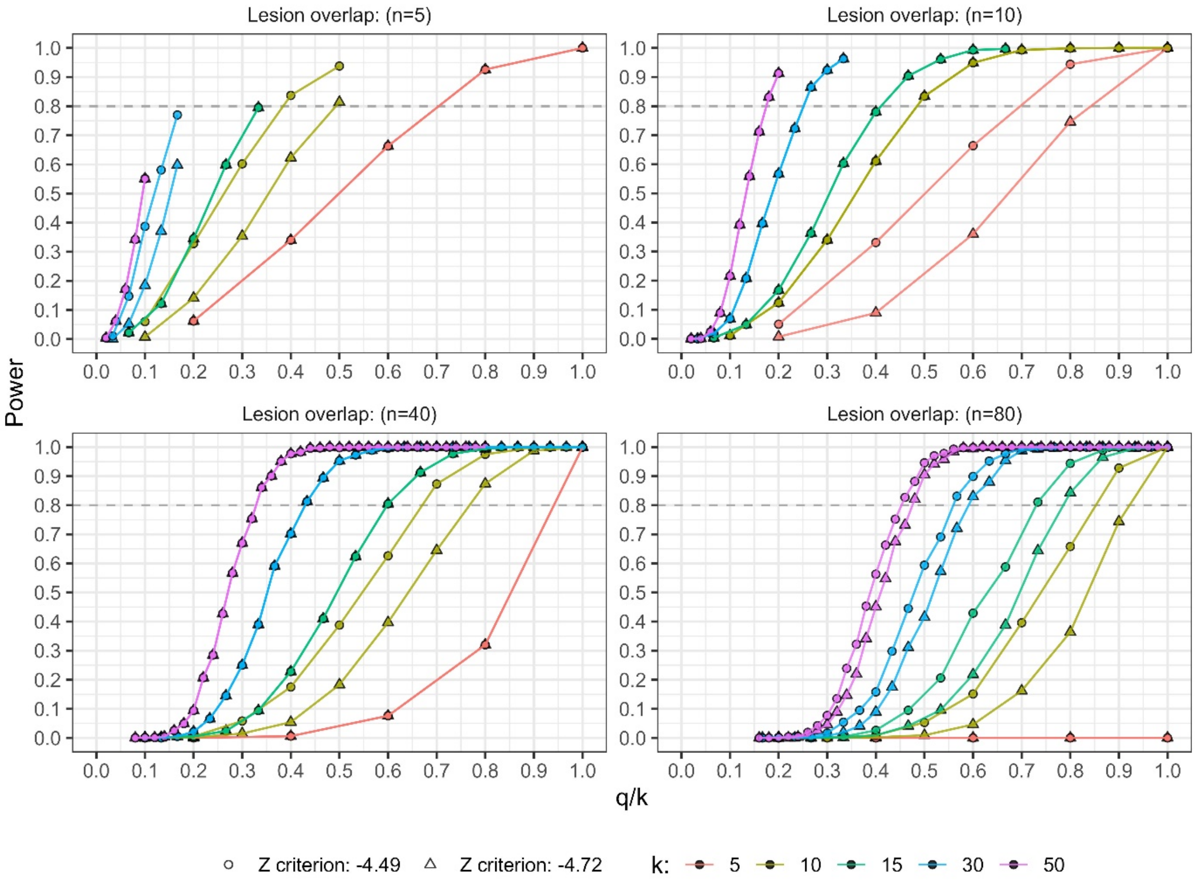

Figure S3.2. Power curves for VLSM. The achieved power for different contingency tables characterized by a number of patients with behavioural impairment (k), number of patients with lesion overlap in the voxel (n) and proportion of patients with behavioural impairment and a lesion relative to number of patients with behavioural impairment (q/k) for the two critical z-scores.

Table S3.2. Odds ratios and (achieved power) for different sample sizes of behavioural impairment and lesion overlap.

		Lesion overlap (n)						
		**5**	**10**	**20**	**30**	**40**	**50**	**80**
Behavioural impairment (n)	**5**	>1000 (.95)	>1000 (1.00)	>1000 (1.00)	>1000 (1.00)	>1000 (1.00)	>1000 (1.00)	*
	**10**	>1000 (.85)	100 (.82)	53 (.83)	49 (.84)	59 (.88)	44 (.88)	>1000 (1.00)
	**15**	*	145 (.91)	46 (.88)	34 (.90)	31 (.92)	23 (.81)	25 (.84)
	**30**	*	88 (.85)	24 (.82)	17 (.82)	13 (.84)	11 (.82)	10 (.83)
	**50**	*	102 (.82)	18 (.80)	13 (.86)	10 (.86)	9 (.83)	7 (.82)

Table Note. * = 80% power not achieved.

All estimates for power and odds ratios are for a critical z-score = -4.72.

Conclusion

Given the sensitivity analysis, we only performed the VLSM for the behavioural classes for which at least 15 patients had a lesion map and for which there was overlap of lesions in at least 10 patients. The sensitivity analysis revealed that the minimal odds ratio that we can detect with more than 80% power given these criteria equals 7. Small odds ratios can thus not be detected.

Results of VLSM and atlas comparison

Resultant significant voxels clusters (>10 contiguous voxels) were compared to anatomical atlases using the Python package AtlasReader (Notter et al., 2019). Neuroanatomical data were visualized using nilearn (Abraham et al., 2014).

5-class solution

Table S3.3. Clusters of significant voxels, their mean intensity, volume and overlap with anatomical regions from three atlases for the 5-class solution.

	MI	Vol (mm^3)	AAL atlas	Desikan-Kiliany atlas	Harvard Oxford atlas
**Class 2**					
1	-5	1502 48	23.35% no label<br>11.26% Temporal Sup R<br>9.05% Temporal Mid R<br>7.11% Insula R<br>6.99% Angular R<br>6.08% Rolandic Oper R	46.31% R CWM<br>17.59% Unknown<br>5.64% R inferior parietal	10.86% R LOC superior division<br>7.97% R Insular Cortex<br>6.28% R Angular Gyrus<br>5.92% R Central Opercular Cortex<br>5.62% R Middle Temporal Gyrus temporo-occipital part
2	-4.54	128	62.50% Frontal Inf Oper R<br>18.75% Frontal Inf Tri R<br>18.75% no label	93.75% R CWM<br>6.25% R rostral middle frontal	100.00% R Middle Frontal Gyrus
**Class 3**					
1	-4.94	1628 0	36.17% Insula L<br>31.25% no label<br>11.45% Putamen L<br>7.62% Frontal Inf Oper L	45.11% L CWM<br>17.25% L Insula<br>17.25% Unknown<br>12.73% L Putamen	38.48% L Insular Cortex<br>17.69% L Putamen<br>11.15% L Central Opercular Cortex<br>7.62% no label<br>7.57% L Frontal Operculum Cortex

					7.37% L Precentral Gyrus
2	-4.63	352	100.00% Precentral L	45.45% Unknown<br>36.36% L precentral<br>18.18% L CWM	68.18% L Precentral Gyrus<br>31.82% L Middle Frontal Gyrus
3	-4.67	240	100.00% Frontal Inf Tri L	40.00% Unknown<br>36.67% L rostral middle frontal<br>23.33% L CWM	100.00% L Middle Frontal Gyrus
4	-4.79	112	100.00% no label	100.00% L CWM	42.86% no label<br>35.71% L Precuneus<br>21.43% L Lateral Ventrical

Table S3.4. Coordinates and peak intensity of peak values and their corresponding anatomical labels for the 5-class solution.

class	cluster	x	y	z	PI	AAL	Desikan-Kiliany	Harvard Oxford
2	1	46	-4	2	-7.70	Insula R	Unknown	21.0% R Central Opercular Cortex<br>20.0% R Insular Cortex<br>8.0% R Heschl's Gyrus<br>6.0% R Planum Polare
	2	30	14	30	-4.83	Frontal Inf Oper R	R CWM	10.0% R Middle Frontal Gyrus
3	1	-32	4	8	-6.69	Insula L	L CWM	39.0% L Insular Cortex
	2	-46	0	42	-5.08	Precentral L	L CWM	49.0% L Precentral Gyrus<br>10.0% L Middle Frontal Gyrus
	3	-40	24	26	-5.21	Frontal Inf Tri L	L rostral middle frontal	39.0% L Middle Frontal Gyrus<br>9.0% L Inferior Frontal Gyrus pars triangularis
	4	-28	-60	18	-5.08	no label	L CWM	0% no label

13-class solution

Table S3.5. Clusters of significant voxels per class, their mean intensity (average z-score), volume and their overlap with anatomical regions from three atlases for the 13-class solution.

	MI	***Vol (mm³)***	***AAL atlas***	***Desikan-Kiliany atlas***	***Harvard Oxford atlas***
			**<u>Class 3</u>**		
1	-4.94	70,144	24.68% no label<br>15.07% Calcarine R<br>14.30% Temporal Mid R<br>10.52% Lingual R<br>7.66% Temporal Inf R	58.90% R CWM<br>10.66% Unknown	11.25% R LOC inferior division<br>10.62% R Middle Temporal Gyrus posterior division<br>10.13% R Intracalcarine Cortex<br>8.03% R Lingual Gyrus<br>7.04% R Middle Temporal Gyrus temporooccipital part<br>6.50% R Occipital Fusiform Gyrus

			7.44% Temporal Sup R		5.54% R Occipital Pole
2	-4.42	864	55.56% no label 27.78% Frontal Inf Oper R 15.74% Frontal Mid 2 R	58.33% R CWM 22.22% R caudal middle frontal 16.67% Unknown	37.04% no label 31.48% R Middle Frontal Gyrus 24.07% R Precentral Gyrus 6.48% R Inferior Frontal Gyrus pars opercularis
3	-4.49	320	75.00% no label 12.50% Amygdala R 10.00% Hippocampus R	40.00% R Amygdala 30.00% R Ventral DC 17.50% R CWM 10.00% R Pallidum	57.50% R Amygdala 40.00% R Pallidum
4	-4.31	288	88.89% Precentral R 11.11% Frontal Inf Oper R	47.22% R CWM 41.67% R precentral 11.11% Unknown	94.44% R Precentral Gyrus 5.56% R Inferior Frontal Gyrus pars opercularis
5	-4.37	288	94.44% Supramarginal R 5.56% Temporal Sup R	72.22% R supramarginal 13.89% Unknown 11.11% R CWM	50.00% R Supramarginal Gyrus anterior division 44.44% R Supramarginal Gyrus posterior division 5.56% R Parietal Operculum Cortex
6	-4.37	272	73.53% Frontal Inf Oper R 17.65% Rolandic Oper R 8.82% Frontal Inf Tri R	52.94% Unknown 29.41% R pars opercularis 14.71% R precentral	79.41% R Inferior Frontal Gyrus pars opercularis 20.59% R Precentral Gyrus

7	-4.49	152	84.21% no label 10.53% Putamen R 5.26% Insula R	100.00% R CWM	52.63% R Insular Cortex 36.84% no label 10.53% R Putamen
8	-4.44	144	100% Hippocampus R	100% R Hippocampus	100 % R Hippocampus
9	-4.36	128	100% Putamen R	100% R Putamen	100 % R Putamen

<u>Class 6</u>

1	-5.13	288 32	31.35% Insula L 30.77% no label 12.35% Frontal Inf Oper L 10.16% Putamen L 5.16% Frontal Inf Tri L	38.40% L CWM 22.23% Unknown 14.73% L insula 11.65% L Putamen 6.44% L pars opercularis	32.55% L Insular Cortex 15.90% L Putamen 10.38% L Inferior Frontal Gyrus pars opercularis 9.54% L Frontal Operculum Cortex 8.66% no label 7.33% L Central Opercular Cortex 5.44% L Precentral Gyrus
2	-4.71	304	100.00% no label	100.00% L CWM	78.95% no label 15.79% L Insular Cortex 5.26% L Central Opercular Cortex

<u>Class 8</u>

1	-4.70	624	61.54% Insula R 38.46% Temporal Sup R	67.95% Unknown 16.67% R insula 11.54% R superior temporal	46.15% R Insular Cortex 29.49% R Heschl's Gyrus 23.08% R Planum Polare

<u>Class 10</u>

1	-4.83	3880	63.30% no label 16.70% Thalamus R 15.46% Putamen R	68.45% R CWM 16.29% R Thalamus Proper 9.48% R Putamen	36.08% R Putamen 31.55% R Thalamus 20.00% no label
2	-4.64	80	60.00% Postcentral R 40.00% no label	40.00% ctx R precentral 40.00% R CWM 20.00% Unknown	70.00% R Precentral Gyrus 30.00% R Postcentral Gyrus
3	-4.53	80	100% no label	90.00% R CWM 10.00% R precentral	100% R Postcentral Gyrus

Class 12

1	-4.48	192	75.00% Heschl L 16.67% Temporal Sup L 8.33% Insula L	50.00% Unknown 20.83% L insula 16.67% L transverse temporal 12.50% L CWM	66.67% L Heschl's Gyrus 33.33% L Insular Cortex
2	-4.57	176	81.82% Temporal Sup L 18.18% Insula L	50.00% Unknown 31.82% L superior temporal 13.64% L CWM	86.36% L Planum Polare 13.64% L Insular Cortex

Class 13

1	-4.54	552	44.93% no label 28.99% Precentral L 21.74% Frontal Inf Oper L	71.01% L CWM 14.49% L caudal middle frontal 10.14% Unknown	73.91% L Precentral Gyrus 15.94% L Middle Frontal Gyrus 5.80% no label

2	-4.56	336	92.86% Frontal Inf Tri L<br>7.14% no label	54.76% Unknown<br>40.48% L pars triangularis	100 % L Inferior Frontal Gyrus pars triangularis
3	-4.56	144	55.56% Insula L<br>44.44% no label	77.78% L insula<br>22.22% L CWM	100 % L Insular Cortex
4	-4.54	120	86.67% Hippocampus L<br>13.33% no label	46.67% L choroid plexus<br>46.67% L CWM<br>6.67% L Hippocampus	100 % L Hippocampus
5	-4.48	104	100.00% Frontal Inf Tri L	76.92% Unknown<br>23.08% L pars opercularis	84.62% L Inferior Frontal Gyrus pars opercularis<br>7.69% L Inferior Frontal Gyrus pars triangularis<br>7.69% L Middle Frontal Gyrus
6	-4.53	96	100.00% no label	66.67% L CWM<br>25.00% L bankssts<br>8.33% Unknown	58.33% L Middle Temporal Gyrus posterior division<br>25.00% no label<br>16.67% L Superior Temporal Gyrus posterior division
7	-4.56	88	100.00% no label	100% L CWM	90.91% no label<br>9.09% L Hippocampus

Note. MI = cluster mean intensity, Vol = lesion volume, LOC = Lateral Occipital Cortex, CWM = Cerebral White Matter.

Table S3.6. Coordinates and peak intensity of peak values and their corresponding anatomical labels for the 13-class solution.

class	***cluster***	***x***	***y***	***z***	***PI***	***Atlas comparison***		
						AAL	***Desikan-Kiliany***	***Harvard Oxford***
	1	24	-80	6	-6.92	/	R CWM	/
	2	32	-6	26	-4.86	/	R CWM	/
	3	20	-6	-10	-5.06	/	R Amygdala	44% R Amygdala
	4	56	4	32	-4.43	Precentral R	R CWM	43% R Precentral Gyrus
3	5	66	-36	30	-4.34	SupraMarginal R	R supramarginal	45% R Supramarginal Gyrus posterior division
	6	60	16	6	-4.63	Frontal Inf Oper R	/	31% R Inferior Frontal Gyrus pars opercularis
	7	32	-2	14	-4.68	/	R CWM	7% R Insular Cortex
	8	30	-18	-16	-4.99	Hippocampus R	R Hippocampus	99% R Hippocampus
	9	26	-4	10	-4.55	Putamen R	R Putamen	98% R Putamen

6	1	-30	6	12	-7.45	Insula L	L CWM	23% L Insular Cortex
	2	-30	-20	26	-5.32	/	L CWM	/
8	1	48	-10	0	-5.18	Temporal Sup R	/	31% R Heschl's Gyrus 24% R Planum Polare
10	1	22	-18	18	-5.82	/	R CWM	/
	2	44	-10	32	-5.15	Postcentral R	/	40% R Precentral Gyrus
	3	44	-16	32	-4.71	/	R CWM	21% R Postcentral Gyrus
12	1	-42	-20	8	-4.85	Heschl L	L transverse temporal	67% L Heschl's Gyrus
	2	-44	-10	-6	-5.09	Temporal Sup L	/	58% L Planum Polare
13	1	-34	2	22	-4.96	/	L CWM	/
	2	-58	30	10	-4.89	/	/	14% L Inferior Frontal Gyrus pars triangularis
	3	-36	6	-4	-4.96	/	Ctx L insula	54% L Insular Cortex

4	-36	-22	-12	-4.81	Hippocampus L	L CWM	29% L Hippocampus
5	-58	20	24	-4.35	Frontal Inf Tri L	/	18% L Inferior Frontal Gyrus pars opercularis
6	-40	-40	2	-4.72	/	L CWM	/
7	-40	-36	-6	-4.44	/	L CWM	/

Table Note. MNI coordinates belonging to a certain ROI per atlas (Harvard-Oxford: highest probable ROI).
PI = peak intensity. / = MNI coordinate not labelled in atlas.

Association of cognitive profiles and functional dysconnectivity

Method

To compare the functional dysconnectivity networks of patients assigned to a class versus all other patients, we first estimated the networks for each patient using the Matlab Lesion Quantification Toolkit (Griffis et al., 2021b). This results in a disconnection matrix containing 135 parcels (regions of interest) and 9045 unique edges (i.e., a disconnection between a pair of two parcels). *This matrix was reduced to only contain the edges that were disconnected in at least 1 patient (1375 edges).*

Then, the linear ANOVA model in the R package NBR (Gracia-Tabuenca & Alcauter, 2020) was used to compare the disconnection matrices of class members to other participants. We tested for differences between class members and others for each edge, assuming that edges that differ between groups are clustered into components (i.e., a subnetwork of connected edges). The latter allows for more statistical power than mass-univariate statistical comparisons (Kim et al., 2014; Zalesky et al., 2010).

To identify suprathreshold edges, a p-value threshold of .01 was used. There are no clear guidelines on which threshold to use to identify suprathreshold edges (Kim et al., 2014; Zalesky et al., 2010). For this reason, we checked the differences in edge weights for edges that were part versus not part of the significant component of edges to check how conservative our analysis was (Figure S3.3). Significant components of edges were identified with the family-wise error p-value for the sum of edge weights of the component, estimated with 1000 permutations.

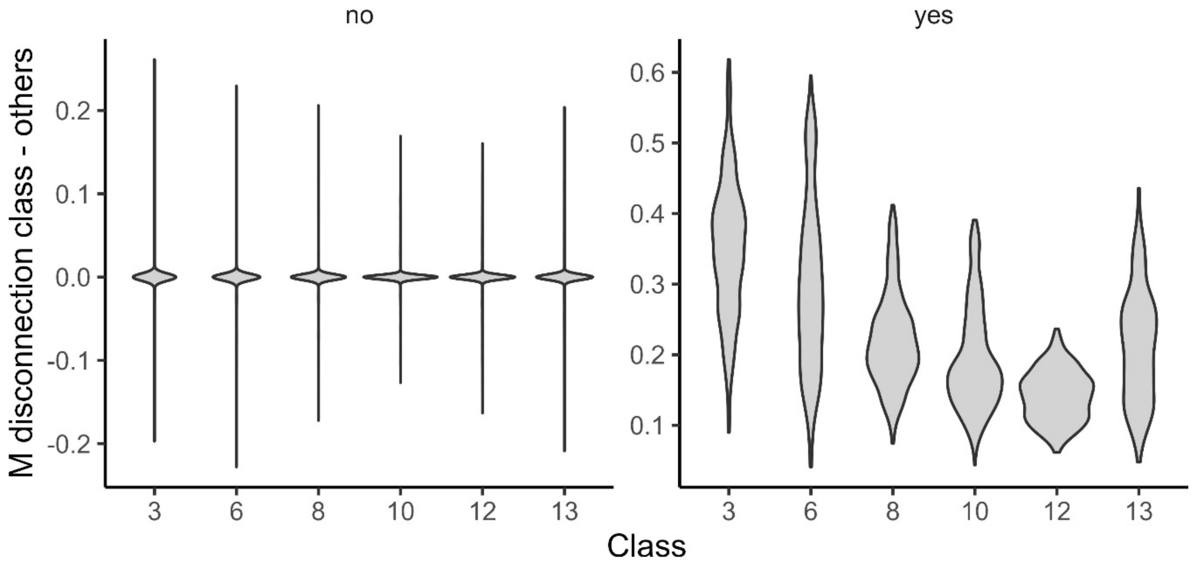

Figure S3.3. Differences in average disconnections between class members and others for edges not included in the significant network (left panel) and edges included in the significant network (right panel) for each behavioural class for which a significant subnetwork emerged.

Results

For the 5-class solution, a statistically significant subnetwork of disconnections was identified for Class 1, 2, and 3 which consisted of disconnections that were more disconnected in Class members versus all others (Table S3.7). For Class 4, significant subnetwork was identified, but this subnetwork only consisted of disconnections which were less severe in Class 4 members than other patients. Last, for Class 5 not a single significant subnetwork was identified.

For the 13-class solution, a statistically significant subnetwork of disconnections was identified for the right-lateralized classes (i.e., Class 3, 8, and 10), but not for Class 9. For the left-lateralized classes a significant subnetwork was identified for Classes 6, 12 and 13. For the non-lateralized classes, no significant subnetwork consisting of connections that were more disconnected in class members versus others was identified (i.e., Class 2, 4, and Class 7). The other classes (Class 1, 5, and 11) were excluded from the network analysis due to an insufficient number of patients with a lesion map (< 15 patients).

Table S3.7. Results of the two-sided NBR test

Class	Component	FWE corrected	Number of significant disconnections

		p-value edge weights	Total	Class > Others
		**5 Class Solution**		
1	1	< .001	534	233
2	1	< .001	1006	773
3	1	< .001	550	429
4	1	< .001	425	0
	2	.55	0	0
	3	.75	0	0
5	1	.003	156	0
	2	.29	0	0
	3	.03	0	0
	4	.60	0	0
	5	.75	0	0
		**13 Class Solution**		
2	1	.29	0	0
3	1	< .001	615	360
4	1	< .001	569	0
	2	.77	0	0
6	1	< .001	419	360
7	1	.135	0	0
8	1	< .001	505	476
9	1	.41	0	0
	2	.28	0	0
	3	.79	0	0
	4	.70	0	0
	5	.41	0	0
10	1	< .001	612	493
12	1	< .001	292	186
13	1	< .001	394	266

To gain insight in which functional disconnections were most characteristic of a class, we further inspected the edges which were more disconnected in Class members versus others. We quantified network disconnection as the number of edges which were significantly more disconnected relative to all network edges. An edge was considered part of a functional network if at least one node belonged to the network. Thus, disconnections between two nodes of a functional network and disconnections between a node of a functional network with nodes of other networks are taken into consideration. This approach provides insight in the relative contributions of each functional network for Class membership.

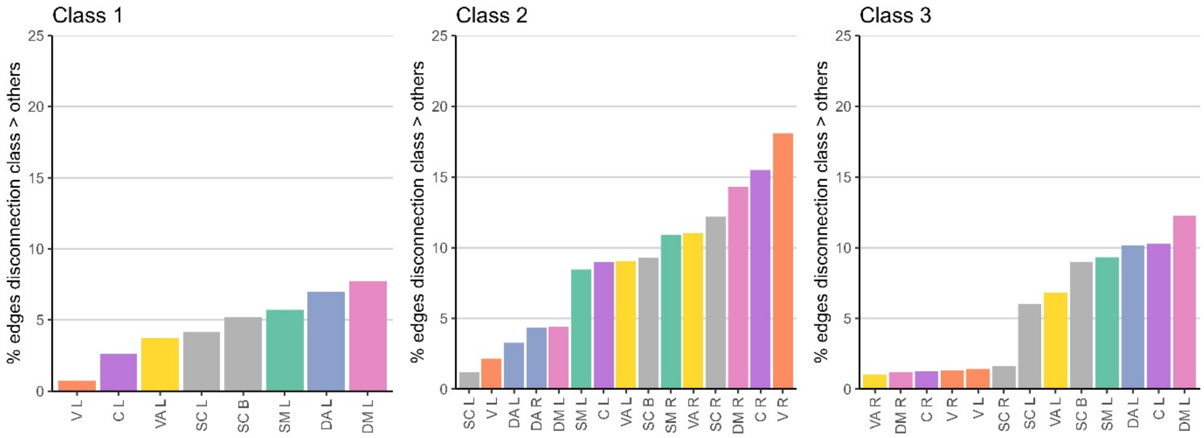

Figure S3.4. Relative network contributions per class of the 5-class solution for which significant disconnections were identified.

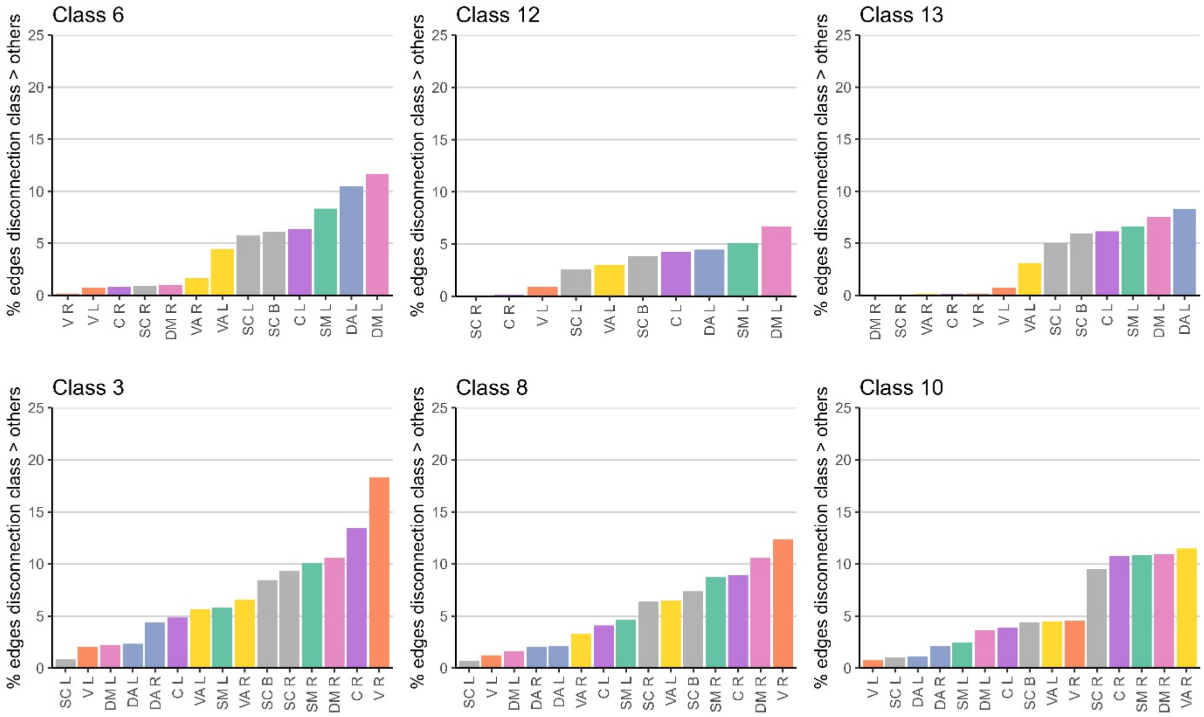

Figure S3.5. Relative network contributions per class of the 13-class solution for which significant disconnections were identified.

Multivariate Similarity Analysis

Method

To quantify behavioural similarity, we computed the Manhattan distances between each pair of participants on the class-probability vectors (which are normalized to sum to 1 per participant) extracted from the LCA model.

To quantify neuroanatomical similarity, several metrics were used. Similarity in lesion location at the voxel-level (i.e., lesion overlap) was quantified using the Dice coefficient for each pair of participants. The difference between lesion volume for each pair of participants was also computed.

In addition, neuro-anatomical similarity at the level of disconnections (tract-based and functional disconnections) was quantified. The disconnections were first standardized. Standardizing ensures that features (disconnected tracts or edges) have equal relative weights when computing the distances between each pair of participants. Then, the Manhattan distance metric was calculated for each pair of participants for both matrices separately. The distance metric represents how dissimilar participants are in their disconnections.

We then assessed the pairwise Spearman associations of the behavioural distances with each neuro-anatomical distance (lesion location, lesion volume, and tract and functional disconnections) using the Mantel Test in the R package ecodist (Goslee & Urban, 2007), which considers the non-independence of the distances.

The Mantel Test was estimated for the full sample and for subsets of patients to assess the potential moderating effect of time after stroke, stroke type (ischemic versus haemorrhagic stroke) and severity of premorbid brain health (Fazekas and GCA ratings).

In addition, to assess the potential impact of influential cases, we estimated the associations across subsets of data leaving out one case at a time. This check did not reveal influential observations.
